# Spider Silk‐Inspired Conductive Hydrogels for Enhanced Toughness and Environmental Resilience via Dense Hierarchical Structuring

**DOI:** 10.1002/advs.202500397

**Published:** 2025-02-04

**Authors:** Seokkyoon Hong, Jiwon Lee, Taewoong Park, Jinheon Jeong, Junsang Lee, Hyeonseo Joo, Juan C. Mesa, Claudia Benito Alston, Yuhyun Ji, Sergio Ruiz Vega, Cristian Barinaga, Jonghun Yi, Youngjun Lee, Jun Kim, Kate J. Won, Luis Solorio, Young L. Kim, Hyowon Lee, Dong Rip Kim, Chi Hwan Lee

**Affiliations:** ^1^ Weldon School of Biomedical Engineering Purdue University West Lafayette IN 47907 USA; ^2^ School of Mechanical Engineering Hanyang University Seoul 04763 Republic of Korea; ^3^ Center for Implantable Devices Purdue University West Lafayette IN 47907 USA; ^4^ Birck Nanotechnology Center Purdue University West Lafayette IN 47907 USA; ^5^ Elmore Family School of Electrical and Computer Engineering Purdue University West Lafayette IN 47907 USA; ^6^ School of Mechanical Engineering Purdue University West Lafayette IN 47907 USA; ^7^ School of Materials Engineering Purdue University West Lafayette IN 47907 USA

**Keywords:** bioinspired materials, environmental resilience, hierarchical structures, tough hydrogels, wearable sensors

## Abstract

Conductive hydrogels, known for their biocompatibility and responsiveness to external stimuli, hold promise for biomedical applications like wearable sensors, soft robotics, and implantable electronics. However, their broader use is often constrained by limited toughness and environmental resilience, particularly under mechanical stress or extreme conditions. Inspired by the hierarchical structures of natural materials like spider silk, a strategy is developed to enhance both toughness and environmental tolerance in conductive hydrogels. By leveraging multiscale dynamics including pores, crystallization, and intermolecular interactions, a dense hierarchical structure is created that significantly improves toughness, reaching ≈90 MJ m⁻^3^. This hydrogel withstands temperatures from −150 to 70 °C, pressure of 12 psi, and one‐month storage under ambient conditions, while maintaining a lightweight profile of 0.25 g cm⁻^3^. Additionally, its tunable rheological properties allow for high‐resolution printing of desired shapes down to 220 µm, capable of supporting loads exceeding 164 kg m⁻^2^. This study offers a versatile framework for designing durable materials for various applications.

## Graphical abstract

Inspired by spider silk, tough and environmentally resilient hydrogels suitable for biomedical sensors, structural supports, protective equipment, and additive manufacturing have been developed.

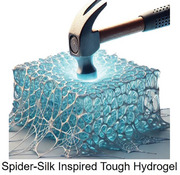

## 1 Introduction

Hydrogels, which are 3D polymeric networks saturated with water, hold great promise for biomedical devices due to their biocompatibility, mechanical softness, and responsiveness to external stimuli.^[^ ^1^ ^]^ However, their high water content weakens molecular chain interactions, resulting in insufficient toughness, limiting their utility in structural components and impact‐resistant applications.^[^ ^2^ ^]^ Poly(vinyl alcohol) (PVA)‐based hydrogels have emerged as a promising platform for achieving toughness due to their ability to form physically cross‐linked networks via hydrogen bonding and crystallization.^[^ ^3^ ^]^ Traditional strategies to enhance hydrogel toughness often focus on molecular engineering of polymer chain interactions in hydrogel framework.^[^ ^4^ ^]^ A common approach involves double‐network hydrogels, where an interpenetrating polymer network is grown within a secondary network to dissipate energy efficiently during deformation.^[^ ^4^ ^]^ Despite these advances, even well‐designed double‐network hydrogels struggle to achieve toughness exceeding 10 MJ m^−3^ due to the limited structural changes occurring within a narrow length scale. Most hydrogels rely on enhancing toughness at a single scale, lacking comprehensive analysis across multiple scales. High toughness, along with high mechanical strength, is desirable for withstanding mechanical stresses, which is crucial for applications in wearable devices, soft robotics, and implantable electronics that require durable materials.^[^ ^5^ ^]^

Recent design principles have been explored to achieve high toughness by employing photo‐crosslinking and interfacial bonding to create soft‐hard material interface microstructures,^[^ ^6^ ^]^ salting‐out treatments to develop ordered‐to‐disordered structures,^[^ ^7^ ^]^ solvent exchange to develop adaptable crystalline domains^[^ ^8^ ^]^ and strong hydrogen bonding,^[^ ^9^ ^]^ annealing to enhance crystallinity,^[^ ^10^ ^]^ and mechanical training to align hydrogel fibers.^[^ ^11^ ^]^ However, challenges remain in achieving environmental tolerance in most hydrogels, as their high water content (50‐95 wt.%) can result in a loss of flexibility and conductivity, especially in low‐temperature and dry environments.^[^ ^12^ ^]^ Improving the environmental resilience of conductive hydrogels is essential for their broader use across a range of demanding applications.^[^ ^13^ ^]^

Natural materials such as spider silk are known for being lightweight yet exceptionally strong, tough, and environmentally resilient, primarily due to their hierarchical structures that span multiple length scales.^[^ ^14^ ^]^ These structures are composed of organized layers and components at multi‐scales, which contribute to their impressive mechanical properties. For example, the toughness of spider silk, which reaches 150 MJ m⁻^3^, is attributed to its hierarchical structure, featuring nanocrystalline domains and extensive hydrogen bonding.^[^ ^14^, ^15^ ^]^ These complex architectures significantly enhance both the overall toughness of the material and its ability to withstand environmental stress. By mimicking the hierarchical assembly of multiscale architectures in hydrogels, including molecular, nano, and micro scales, we can similarly improve their toughness and environmental resilience. This approach effectively translates the design principles of nature's most durable materials into the creation of robust conductive hydrogels, potentially expanding their applications and improving their performance in various conditions.

Here, we developed tough, environmentally tolerant, and conductive hydrogels by designing unique dense hierarchical structures inspired by spider silk, leveraging multiscale dynamics such as pores, crystallization, and intermolecular interactions. Our hydrogels exhibit impressive toughness, reaching ≈90 MJ m⁻^3^, and demonstrate environmental tolerance across a wide range of conditions, from minus 150 to 70 °C, under 12 psi, and during one‐month storage in ambient conditions—a combination of attributes that is rarely achieved. These exceptional characteristics result from three key factors: 1) the formation of dense structures at the microscale, 2) enhanced crystallinity and increased crystallite size at the nanoscale, and 3) strengthened hydrogen bonding at the molecular level. Additionally, our hydrogels exhibit a low water content, <32.5 wt.%, which contributes to their stability and performance in challenging environments. With controlled rheological properties, these hydrogels can be printed through a dispenser into various patterns, such as spider webs. For example, a printed tough hydrogel, shaped into specific forms, can support the weight of a 70 kg person and protect delicate laboratory equipment and glassware from the impact of a 50 g dropping weight. It can also withstand mechanical impacts from a dropping ball weighing over 164 kg m⁻^2^ without mechanical failure. This study offers fundamental insights into the engineering of dense hierarchical structures, using multiscale dynamics of pores, crystallization, and intermolecular interactions to transform inherently weak hydrogels into tough, environmentally resilient, and lightweight materials. These advancements greatly expand the potential applications of hydrogels, moving beyond biomedical sensors to include structural support, protective equipment, and additive manufacturing, thereby opening new opportunities across various industries.

## 2 Results and Discussion

### 2.1 Dense Hierarchical Structuring for Improved Hydrogel Toughness

**Figure 1:**
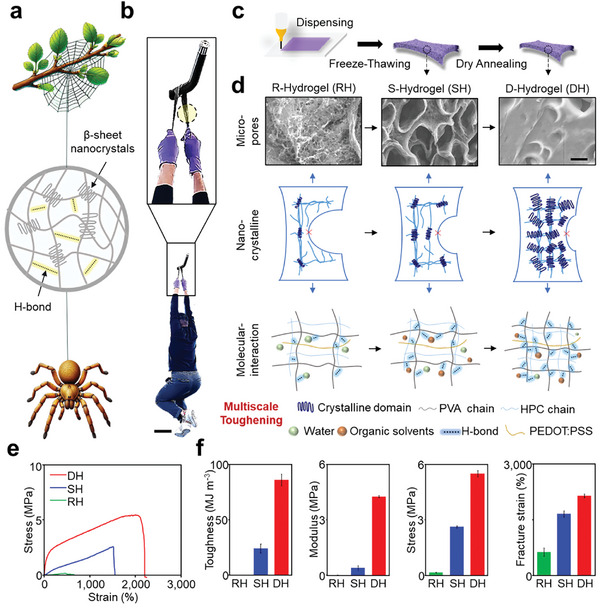
Spider silk‐inspired hydrogel toughened via dense hierarchical structuring. a) Schematic illustration of the hierarchical structure of spider silk. b) DH, supporting the weight of a 70 kg man. Scale bar, 25 cm. c) Schematic illustration of the fabrication process of DH. d) Multiscale toughening via dense hierarchical structuring. Scale bar, 40 µm. e) Stress‐strain curve of RH, SH, and DH. f) A comparison of toughness, modulus, stress, and fracture strain of RH, SH, and DH.

The remarkable toughness of spider silk stems from its hierarchical structure, composed of β‐sheet nanocrystals and strong hydrogen bonding (**Figure** **1**a).^[^ ^14^, ^15^ ^]^ Inspired by the natural design of spider silk such as nanocrystalline and hydrogen bonding, we developed tough hydrogels through solvent engineering (S‐Hydrogel, SH) and dry‐annealing (D‐Hydrogel, DH) of a regular hydrogel (R‐Hydrogel, RH). These processes promote dense hierarchical structuring by enhancing 1) the overall density of the structure, 2) crystallinity and crystallite size, and 3) hydrogen bonding. As a result, the D‐Hydrogel can support the weight of a 70 kg person, enduring more than 3000 times their own weight without mechanical failure (Figure 1b; Movie S1, Supporting Information). D‐Hydrogels were synthesized through a combination of dispensing, freeze‐thawing, and dry‐annealing processes (Figure 1c). A PVA‐based hydrogel was employed as a model system due to its tunable microstructure, crystallinity, and intermolecular interactions. Hydroxypropyl cellulose (HPC) was introduced as a short‐chain additive to strengthen the mechanical properties of the hydrogel by enhancing the matrix reinforcement. Poly(3,4‐ethylenedioxythiophene):polystyrene sulfonate (PEDOT:PSS) was selected for its excellent electrical conductivity and aqueous processability.^[^ ^16^ ^]^ Ethylene glycol prevents rigidification and freezing at low temperatures through strong hydrogen bonding with water.^[^ ^17^ ^]^ Glycerol's hydration retention prevents water volatilization and enhances long‐term stability, forming hydrogel networks more stable than those with water.^[^ ^18^ ^]^ The synthesis process involved vigorous mechanical mixing, dispensing, and freeze‐thawing to generate physically cross‐linked hydrogels, with solvents tuning the structures across multiple scales. Subsequent dry annealing of the hydrogels resulted in the formation of dense structures within the hydrogels. The absence of new characteristic peaks in the hydrogels indicates that no new chemical bonds were formed between PVA and HPC (Figure S1, Supporting Information). To investigate the factors contributing to hydrogel toughness, we prepared three types of hydrogels (RH, SH, and DH) with varying toughness levels of 0.5, 24, and 86 MJ m⁻^3^ (Figure S2, Supporting Information). Toughening mechanisms are analyzed across multiple scales, from micro to molecular levels, providing insights into how hierarchical structuring enhances hydrogel toughness (Figure 1d).

At the microscale (>1 µm), scanning electron microscopy (SEM) reveals changes in pore architecture within the hydrogels (Figure 1d, top row and Figure S3, Supporting Information). During formation, large ice crystals created by the freezing process push polymer chains together, resulting in a porous structure. R‐Hydrogels, containing ≈81 wt.% water, demonstrate a highly porous architecture with numerous pores and fine backbones. When 48.7 wt.% organic solvents are incorporated and the water content is reduced to 32.5 wt.%, these highly porous structures with thin backbones transform into denser porous structures with thicker backbones, as seen in the S‐Hydrogel, due to increased intermolecular interactions.^[^ ^19^ ^]^ Dry annealing further promotes tighter molecular chain alignment, reduces water content to below 32.5 wt.%, and achieves the 34% volume shrinkage with pore closure, thereby enhancing the toughness of the hydrogel (Figure S4, Supporting Information).^[^ ^20^ ^]^

At the nanoscale (from 1 nm to 1 µm), the toughening process is driven by increased crystallinity and crystallite size, as identified through X‐ray diffraction (XRD) and differential scanning calorimetry (DSC) (Figure 1d, middle row, Figures S5 and S6, Supporting Information). XRD results show a sharp peak at 2θ ≈ 19.4°, corresponding to the intermolecular interference between the polyvinyl alcohol (PVA) chains and the diffraction of the PVA crystal plane^[^ ^11^ ^]^ (Figure S5, Supporting Information). The R‐Hydrogel initially has the lowest crystallinity and crystallite size. However, the introduction of organic solvents increases both crystallinity and crystallite size, leading to the formation of the S‐Hydrogel. This suggests that PVA chains have greater mobility in the supercooled state, allowing them to form more ordered structures over a larger area.^[^ ^21^ ^]^ Dry annealing further enhances crystallinity and crystallite size by increasing the mobility and regular arrangement of PVA chains, along with the formation of additional hydrogen bonds.^[^ ^22^ ^]^ Furthermore, DSC analysis shows that the area of the endothermic melting peaks of the D‐Hydrogel is larger compared to the S‐Hydrogel and R‐Hydrogel, signifying a greater number of crystalline domains in the D‐Hydrogel (Figure S6, Supporting Information). These crystalline domains help delay chain fracture by crack‐pinning, which further enhances toughness.^[^ ^23^ ^]^

On the molecular scale (< 1 nm), the toughening mechanism is illustrated by the dynamics of intermolecular and intramolecular interactions, such as hydrogen bonding, as shown by Fourier transform infrared (FTIR) spectroscopy (Figure 1d, bottom row, Figure S7, Supporting Information). The FTIR spectrum of the R‐Hydrogel shows characteristic stretching bands of C─O at 1091 cm^−1^, while these bands shift to 1038 and 1033 cm^−1^ for the S‐Hydrogel and D‐Hydrogel, respectively. The shift to lower wave numbers indicates the formation of stronger hydrogen bonds within the hydrogel.^[^ ^24^ ^]^ The organic solvents strongly interact with PVA chains, enhancing the hydrogel's mechanical properties. After dry annealing, the absorbance of the intermolecular order (crystal‐sensitive band at 1143 cm^−1^) of PVA becomes more intense, suggesting that annealing not only enhances PVA crystallization but also strengthens intermolecular interactions between PVA molecular segments.^[^ ^25^ ^]^ These enhanced interactions contribute to the toughness of hydrogels at the molecular level.

As a result, the combination of pore closure, increased crystallinity and crystallite size, and stronger hydrogen bonding significantly improves toughness, mechanical strength, fracture strain, and modulus, surpassing the properties of previously developed tough hydrogels (Figure 1e,f; Figure S8; Table S1, Supporting Information).^[^ ^10^, ^20^, ^26^ ^]^

### 2.2 Strategies for Improving Hydrogel Environmental Stability

**Figure 2:**
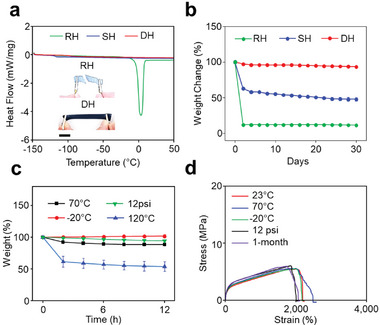
Environmental tolerance. a) DSC curves of RH, SH, and DH. Inset: Images of stretching RH and DH after freezing. Scale bar, 4 cm. b) Weight change of RH, SH, and DH for 1‐month storage. c) Weight change of DH under various environments. d) Stress‐strain curves of DH under various environments.

The limited environmental stability of hydrogels is a major challenge that restricts their adaptability to varying conditions. Enhancing the environmental tolerance of hydrogels is therefore essential for expanding their use across diverse applications.^[^ ^13^, ^27^ ^]^ D‐Hydrogels achieve superior environmental stability through dense hierarchical structuring. For instance, D‐Hydrogels exhibit remarkable anti‐freezing properties due to strong intermolecular interactions. Differential scanning calorimetry (DSC) measurements were conducted over a temperature range from −150 to 50 °C (**Figure** **2**a). A sharp peak at 3.2 °C in R‐Hydrogels corresponds to the freezing point of water within the hydrogel matrix. In contrast, a weak endothermic peak at −116 °C for the S‐Hydrogel indicates the glass transition temperature of the solution, with no freezing point observed in the DSC curve, attributed to enhanced intermolecular interactions between organic solvents and water.^[^ ^28^ ^]^ Notably, no significant peaks were observed in the D‐Hydrogel, attributed to the enhanced intermolecular interactions after the dry annealing process. The D‐Hydrogel retains its stretchability at −20 °C, while R‐Hydrogels lose elasticity and become prone to breakage when frozen at the same temperature (inset in Figure 2a). This indicates that D‐Hydrogels have superior freezing resistance compared to previous hydrogels developed by other methods (Figure S9, Supporting Information).^[^ ^12^, ^29^ ^]^

The anti‐drying capability of hydrogels was tested by exposing them to air at room temperature for up to 30 days, with weight changes monitored throughout this period. The results showed that D‐Hydrogels experienced only minimal weight loss, demonstrating excellent resistance to drying over 30 days due to their enhanced intermolecular interactions and dense structures (Figure 2b). In comparison, the R‐Hydrogel and S‐Hydrogel showed significant weight reduction, indicating severe water loss when exposed to air at room temperature. Further tests on D‐Hydrogels under various conditions, including different temperatures (−20 and 70 °C) and vacuum (12 psi), revealed negligible weight variation over 12 h. This confirms their exceptional environmental tolerance, attributed to their dense structures and strong intermolecular interactions, except when stored at 120 °C (Figure 2c). The D‐Hydrogel began to crumple when the temperature surpassed 120 °C (Figure S10, Supporting Information).

The stress‐strain curve of D‐Hydrogels has similar trends under varying conditions, including low to high temperatures (ranging from −20 to 70 °C), a vacuum (12 psi), and long‐term storage (1‐month storage in ambient conditions) (Figure 2d). D‐Hydrogels exhibit high fracture strain and stress under challenging conditions while maintaining significant toughness and modulus across a wide range of environments, showcasing their environmental resilience (Figure S11, Supporting Information). Overall, the dense hierarchical structuring of D‐Hydrogels not only improves their mechanical properties but also significantly enhances their environmental stability (Figure S12, Supporting Information).

### 2.3 Scalable Fabrication and Multifunctional Capabilities

**Figure 3:**
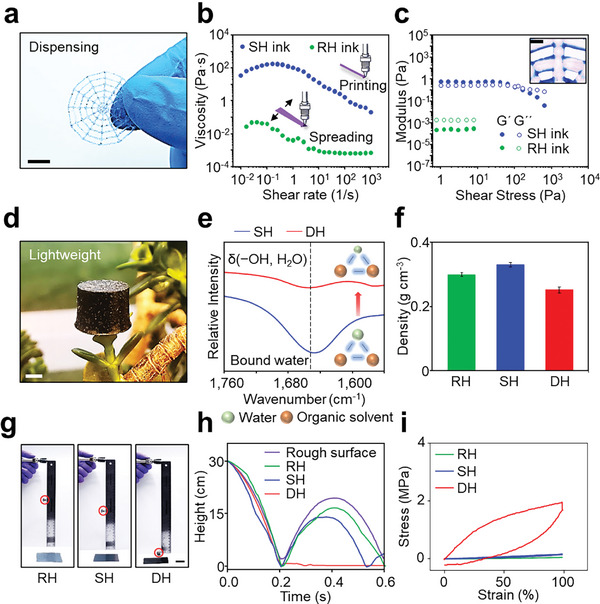
Scalable fabrication and multifunctionality. a) Image of a printed spider web consisting of DH. Scale bar, 1 cm. b) Viscosity as a function of shear rate for RH and SH inks. c) Storage and loss modulus as a function of shear stress for RH and SH inks. Top‐right inset: Image of the printed high‐viscosity SH ink. Scale bar, 500 µm. d) Image of DH, placed on a leaf. Scale bar, 5 mm. e) FTIR of SH and DH. f) Density of RH, SH, and DH. g) Images of free‐falling experiment, showing the rebounding of a steel‐ball onto RH (left), SH (middle), and DH (right). Scale bars, 3 cm h) Height versus time for steel‐ball drop and rebound trails on rough surface, RH, SH, and DH. i) Loading‐unloading curves of RH, SH, and DH subjected to 100% tensile strain.

The design of D‐Hydrogels allows for simple, scalable fabrication from a viscous ink. The high‐viscosity SH ink has favorable rheological properties, making it suitable for printing and creating microstructures of D‐Hydrogels through dispensing (**Figure** **3**a).^[^ ^1a^ ^]^ Figure 3b illustrates the apparent viscosity of both RH and SH inks in relation to shear rate. The RH ink, which uses water as a solvent, shows low viscosity. However, when organic solvents are added to create SH ink, the viscosity increases, making it more suitable for dispensing.^[^ ^30^ ^]^ Figure 3c displays the changes in storage modulus (G′) and loss modulus (G″) for both RH and SH inks under varying shear stress. The RH ink exhibits parallel profiles for G′ and G″ with G″ consistently higher, indicating a liquid‐like behavior. In contrast, the SH ink shows shear‐thinning non‐Newtonian behavior with a yield stress of 101.5 Pa, due to the increased intermolecular interactions from the organic solvents, which enhances its suitability for dispensing (inset in Figure 3c). The D‐Hydrogels produced by dispensing are twistable, foldable, rubbable, and stretchable without losing their material properties (Figure S13; Movie S2, Supporting Information).

Many applications, such as wearables and soft robotics, require low‐density materials, yet hydrogels usually have high density (> 1 g cm^−^^3^) because of their water‐filled polymer matrix.^[^ ^31^ ^]^ D‐Hydrogels uniquely combine lightweight properties with high mechanical strength. Their lightweight nature, achieved through water loss during the dry annealing process, allows the hydrogels to stably rest on a single leaf of a jade plant (Figure 3d). This water evaporation is confirmed by FTIR, which shows a reduction in the peak associated with the bending vibration of hydroxyl (δ (−OH, H_2_O)) at 1647 cm⁻¹, related to bound water (Figure 3e).^[^ ^25^ ^]^ As a result, D‐Hydrogels achieve the lowest density, at 0.25 g cm^−^^3^. While R‐Hydrogels have a high water content (81.2 wt.%) and therefore lower density than S‐Hydrogels, which have a reduced water content (32.5 wt.%), as water is less dense than organic solvents (Figure 3f).

D‐Hydrogels also exhibit excellent shock resistance due to their outstanding energy dissipation capabilities. To demonstrate this, a steel ball was dropped from various heights onto the R‐Hydrogel, S‐Hydrogel, and D‐Hydrogel (Figure 3g). The D‐Hydrogel effectively reduced the rebound height and rapidly stopped the ball, regardless of the initial drop height, demonstrating significant energy dissipation (Figure S14a–d, Supporting Information). In contrast, bare surfaces, R‐Hydrogels, and S‐Hydrogels exhibited noticeable rebound and slower deceleration (Figure 3h; Figure S14e,f; Movie S3, Supporting Information). The superior energy dissipation is attributed to the physically cross‐linked crystalline domains within D‐Hydrogels, which can unzip to release mechanical energy. The high crystallinity and large crystalline domains in D‐Hydrogels enable them to dissipate more mechanical energy than S‐Hydrogels and D‐Hydrogels (Figure 3I; Figure S15, Supporting Information).^[^ ^32^ ^]^ The stability of energy dissipation in D‐Hydrogels is maintained across a variety of conditions (Figure S16, Supporting Information). Additionally, an increasing trend in energy dissipation with higher strain levels has been observed, as more cross‐linking sites are broken, surpassing the performance of previously studied hydrogels (Figure S17, Supporting Information).^[^ ^26a^ ^–d]^

### 2.4 Versatile Sensing Capabilities of D‐Hydrogels

**Figure 4:**
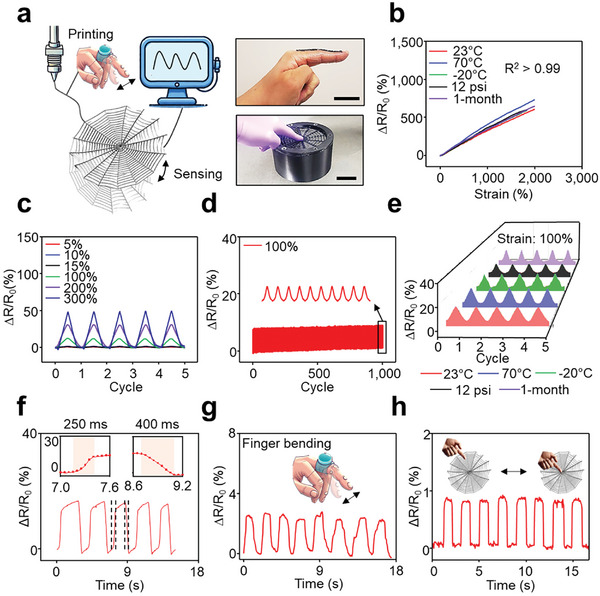
Sensing performance. a) Image of a printed spider web consisting of DH. Scale bar, 1 cm. b) ΔR/R_0_ of DH with strain at various environments. c) ΔR/R_0_ of DH under loading‐unloading cycles at strains of 5%, 10%, 15%, 100%, 200% and 300%. d) ΔR/R_0_ of DH throughout 1000 stretching‐releasing cycles with a strain of 100%. e) ΔR/R_0_ of DH under loading‐unloading cycles at a strain of 100% under diverse conditions. f) Response/recovery time of DH at a strain of 100%. g) Wearable sensing using DH. h) A spider web made of DH for sensing external stimuli.

D‐Hydrogels, with their excellent mechanical properties, environmental tolerance, and unique dispensing and sensing capabilities, are well‐suited for flexible sensing applications (**Figure** **4**a). Figure 4b shows the relative resistance change (ΔR/R_0_) of the tough hydrogel, demonstrating a high degree of linearity (R^2^ > 0.99) for applied strains up to 2000% under various conditions. This indicates a wide electrical sensing range for the D‐Hydrogel, ensuring reliable strain sensing across diverse environments without the need for complex calibration (Figure S18, Supporting Information). Figure 4c illustrates the ΔR/R_0_ response of the D‐Hydrogel during loading‐unloading cycles at multiple strains (5%, 10%, 15%, 100%, 200%, and 300%), revealing consistent response patterns. Furthermore, the D‐Hydrogel maintains exceptional stability and continuous performance under cyclic loading in different conditions (Figure S19, Supporting Information). This stability is maintained over more than 1000 cycles at the strain of 100%, highlighting its robust endurance and sustained performance (Figure 4d). Figure 4e depicts the stable electrical response of the D‐Hydrogel at 100% strain during loading‐unloading cycles across various environments, showcasing its strong durability with low drift. The slight rise in resistance during testing is likely due to the straightening and occasional fracturing of the conductive PEDOT chains under repeated loading and unloading cycles.^[^ ^33^ ^]^ The stable ΔR/R_0_ measurements at different rates (5, 10, 50, 100, and 500 mm min^−1^) further demonstrate a reliable response to various external stimuli in different environments (Figure S20, Supporting Information). When subjected to a 100% strain under diverse conditions, the D‐Hydrogel exhibits consistent response and recovery times, all under 400 ms, indicating no significant deviations (Figure 4f; Figure S21, Supporting Information).

D‐Hydrogels can effectively monitor human movements in real‐time, providing valuable data on joint strain strength, intensity, and consistency.^[^ ^34^ ^]^ This capability has important implications for biomechanics and kinesiology, potentially improving healthcare and human performance by preventing injuries,^[^ ^35^ ^]^ enhancing pain management,^[^ ^36^ ^]^ and enabling precise gait analysis.^[^ ^37^ ^]^ To demonstrate their potential, prototypes were developed to measure strain changes during human motion, such as finger movements (Figure 4g). The D‐Hydrogel showed stable resistance changes in response to body movements. The results of skin irritation tests confirm that D‐Hydrogels are safe for skin contact (Figure S22, Supporting Information). Specifically, the hydrogel and 3M tapes were placed on human skin for ≈ 10 min before being removed, with untreated bare skin used as a control. Notably, no skin irritation was observed following the application of the hydrogel, whereas the 3M tapes caused visible irritation. These results underscore the superior biocompatibility of the hydrogel. Detailed descriptions of the skin irritation tests are provided in the Experimental Section. Additionally, a spider web structure made from the D‐Hydrogel demonstrated excellent sensing abilities in detecting external stimuli, further highlighting its biocompatibility and suitability for use in motion and external stimuli detection sensors (Figure 4h).

### 2.5 Hydrogel‐Based Synthetic Webs for Impact Protection

**Figure 5:**
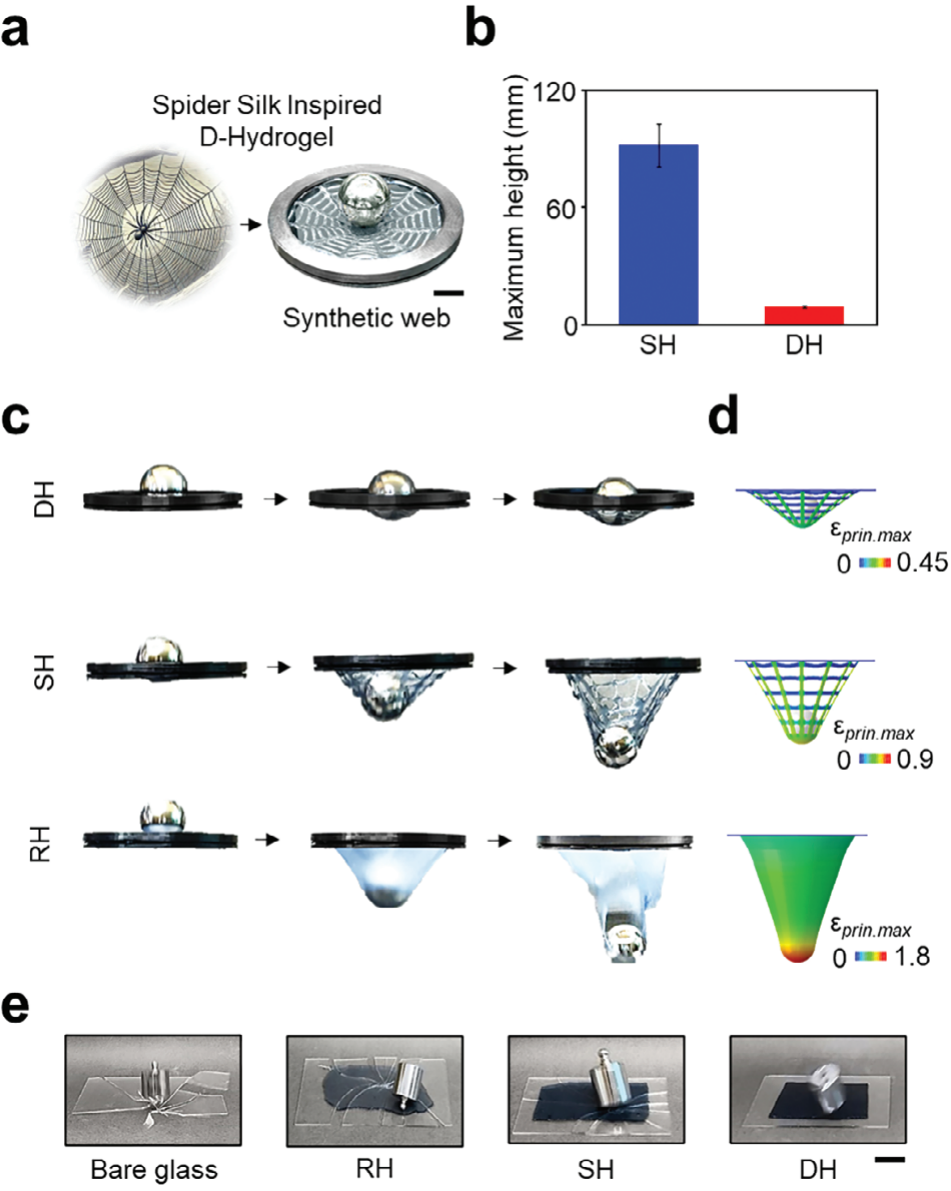
Hydrogel‐based synthetic webs a) A synthetic web made of the D‐Hydrogel inspired by the spider silk. Scale bar, 2 cm. b) Deformation height of SH and DH. Scale bar, 2 cm. c) Snapshots of steel‐ball, impacting RH, SH, and DH. Scale bars, 2 cm. d) FEA results of steel‐ball, impacting RH, SH, and DH. e) Images of RH, SH, and DH used as glass protection during impact. Scale bar, 2 cm.

Spider silk is renowned for its exceptional toughness and environmental tolerance, largely due to its hierarchical structure. Inspired by the toughness of spider silk, we developed a synthetic web made from D‐Hydrogels (**Figure** **5**a). We created two types of synthetic webs using S‐Hydrogels and D‐Hydrogels, as well as a film made from R‐Hydrogels. The S‐Hydrogel ink, with its favorable rheological properties, is suitable for printing and allows the fabrication of synthetic webs using both S‐Hydrogels and D‐Hydrogels through dispensing. In contrast, the R‐Hydrogel ink has low viscosity, making it unsuitable for dispensing.

The maximum deformation height of a synthetic web made from D‐Hydrogel was significantly lower than that of a web made from S‐Hydrogel under the impact of a 225 g steel ball (Figure 5b). The deformation behavior of three types of hydrogels was analyzed through steel ball impact experiments (Figure 5c; Movie S4, Supporting Information). The synthetic web made from D‐Hydrogels demonstrated remarkable mechanical properties, as it could withstand the impact of a steel ball weighing 225 g without noticeable deformation (Figure 5c, top row). While the synthetic web made from S‐Hydrogels could support the same steel ball (225 g) by undergoing considerable deformation due to its relatively lower mechanical strength (Figure 5c, middle row). However, the R‐Hydrogel film was unable to withstand the weight and tore under the same conditions (Figure 5c, bottom row). The corresponding Finite Element Analysis (FEA) supports insights into the deformation behavior under ball impact (Figure 5d; Figure S23, Supporting Information). The R‐Hydrogel film exhibited the highest strain levels, accompanied by significant deformation. The synthetic web fabricated from S‐Hydrogel demonstrated notable deformation, maintaining strain levels lower than those of the R‐Hydrogel film. The synthetic web made from D‐Hydrogel exhibited the lowest strain levels, accompanied by negligible deformation. Under the larger steel ball (371 g) impact, the synthetic web made from S‐Hydrogels deformed significantly and was eventually torn by the steel ball (Figure S24, top row, Supporting Information). A synthetic web made from D‐Hydrogels was able to fully support the larger steel ball (371 g) with minimal deformation (Figure S24, middle row, Supporting Information). Even with heavier steel balls weighing 534 g, the integrity of the D‐Hydrogel web remained intact, showing no significant deformation, which highlights its superior toughness (Figure S24, bottom row, Supporting Information).

The deformation height of the D‐Hydrogel was significantly lower than that of the S‐Hydrogel, making it more effective as a protective layer, as it transferred less impact energy to the underlying surface. To test this, three types of samples were used to protect glass from impact. Glass protected by the D‐Hydrogel remained intact after a free‐falling steel weight, while bare glass and glass protected by R‐Hydrogel and S‐Hydrogel shattered upon impact (Figure 5e; Figure S25; Movie S5, Supporting Information). Additionally, when a standard laboratory beaker was dropped onto the surface of the D‐Hydrogel (Figure S26; Movie S6, Supporting Information), it remained undamaged upon contact. These findings suggest that the D‐Hydrogel offers excellent cushioning by dissipating energy effectively, making it ideal for protecting fragile items in everyday situations.

## 3 Conclusion

This study introduces a new approach to creating hydrogels with exceptional toughness and environmental resilience, inspired by the structure of spider silk, by designing dense hierarchical structures. By strategically manipulating the dynamics at multiple scales—pores, crystallization, and molecular interactions, we achieve significant improvements in the hydrogels' properties. These enhancements are driven by three primary mechanisms: 1) developing compact structures at the microscale, 2) boosting crystallinity and crystallite size at the nanoscale, and 3) reinforcing hydrogen bonds at the molecular scale. As a result, the hydrogels attain a toughness of ≈ 90 MJ m⁻^3^ and demonstrate the ability to endure diverse environmental conditions, such as extreme temperatures (−150 to 70 °C), high pressure (12 psi), and prolonged exposure to ambient conditions. The tunable rheological characteristics further enable precise, high‐resolution printing, making these hydrogels suitable for various applications, including the fabrication of synthetic webs that can support substantial loads and detect external stimuli.

The outcomes of this research deepen our understanding of the multiscale strategies essential for enhancing the toughness and environmental stability of hydrogels. This study provides a flexible framework for designing materials with similar robust properties, applicable in fields such as wearable sensors, soft robotics, and implantable electronics. While the proposed approach shows significant promise, further investigation may be needed to address challenges related to scalability and cost‐effectiveness in large‐scale production, ensuring consistency and uniformity of properties across larger batches. Although the current formulation may face limitations in extreme conditions beyond the tested range, applying these design principles to other materials could help overcome such constraints and drive innovation across sectors like energy, robotics, and healthcare.

In conclusion, our findings provide a robust foundation for the development of hydrogels with enhanced toughness and environmental durability. This work lays the groundwork for future research in material science, offering new possibilities for applications across various disciplines and industries.

## 4 Experimental Section

### Materials

The PVA (Mw: 146000‐186000), HPC, PEDOT:PSS, and glycerol were purchased from Sigma‐Aldrich, and the ethylene glycol was purchased from Fisher Chemical. All reagents were used as received without further purification, and Silicone cover stranded‐core wires and Cu tapes (CST5) were purchased from Adafruit and Digi‐Key.

### Preparation of Hydrogels

D‐Hydrogels were prepared through dispensing, freeze‐thawing, and dry‐annealing. A total of 4 mL of DI water and ethylene glycol were combined with 0.31 g of HPC and heated to 95 °C until fully dissolved. Subsequently, 1 g of PVA was added to the HPC solution and stirred for more than 2 h until homogeneity was achieved. The resulting PVA‐HPC solution was then thoroughly mixed with 1 g of PEDOT:PSS solution over 2 h. Afterward, 2 mL of glycerol was added to the PVA‐HPC‐PEDOT:PSS solution, which was stirred continuously for another 2 h until a homogeneous solution was achieved. To ensure solution uniformity, a QSONICA probe was used for sonication. The probe, immersed in the solution, emitted ultrasonic waves to break up agglomerates and ensure a homogeneous distribution. After sonication, the solution was carefully poured into a pre‐cleaned mold, avoiding the introduction of air bubbles. It was left undisturbed at room temperature for at least 1 min to conform to the mold shape and allow any air bubbles to dissipate. The mold was then sealed with a pre‐cleaned glass cover to maintain sterility and protect the solution during subsequent steps. Before use, the mold and glass cover were cleaned with acetone, isopropanol, and deionized water to prevent contamination. Following this careful molding process, the solution underwent a freeze‐thawing process to enhance its mechanical and electrical properties. The S‐Hydrogel solution was frozen overnight at −20 °C and then thawed at 25 °C for 3 h. After thawing, the specimen was carefully extracted from the mold and trimmed to the desired shape, producing the S‐Hydrogel. The hydrogel contained 1 g of PVA, 10 mL of DI water, and 1 g of PEDOT:PSS. The R‐Hydrogel contained 0.31 g of HPC, 1 g of PVA, 10 mL of DI water, and 1 g of PEDOT:PSS. Both were synthesized using the same procedure. The PVA long‐chain network provides flexibility, while the HPC short‐chain network adds stiffness, with stretching breaking short chains to dissipate energy and enhance toughness. HPC enhanced the mechanical properties of the hydrogel system, but an excessive amount decreased the maximum strain at break, leading us to select 2.5 wt.% of HPC (Figure S27, Supporting Information).

To prepare synthetic webs, the formulated S‐Hydrogel ink was dispensed with the nozzle injection system (Nordson EFD) onto pre‐cleaned glass, following the same procedures.

### Dry Annealing

The dry annealing process was conducted at a temperature between its glass transition (≈35.5 °C) and melting temperature (≈237 °C), as determined by DSC analysis (Figure S6, Supporting Information). Specifically, the dry annealing process was conducted at 60 °C to achieve the structural stabilization, enhanced crystallization and hydrogen bonding. Initially, the hydrogel was placed between two glass substrates to ensure uniform drying and annealed for 1 h. Subsequently, one glass substrate was removed to expose one side of the hydrogel for further annealing, facilitating more even solvent evaporation and densification. The entire process was conducted overnight until the hydrogels have been stabilized to obtain consistent properties. To ensure thorough annealing of the entire specimen, the sample was flipped five times during the process. The criteria for annealing require that the structural integrity remains intact without crumpling, accompanied by consistent volume and weight changes (Figure S28, Supporting Information). During the dry annealing of R‐Hydrogels with an initial water content of 81.2 wt.%, excessive water evaporation, attributed to weak intermolecular interactions, resulted in significant weight loss of 89% and structural crumpling, ultimately compromising their mechanical integrity (Figure S28a, Supporting Information). In contrast, S‐Hydrogels, with a lower water content of 32.5 wt.% and an organic solvent content of 48.7 wt.%, exhibited controlled densification. The dry annealing of S‐Hydrogels resulted in a consistent volume shrinkage of 34% and weight reduction of 43% without structural crumpling, enabled by stronger intermolecular interactions (Figure S28b, Supporting Information). This process resulted in the formation of D‐Hydrogels, which exhibited strong interactions among water, organic solvents, and the polymer matrix. Dry annealing temperatures above 120 °C caused excessive evaporation, leading to crumpled D‐Hydrogels with excessive weight reduction of 75% and compromised mechanical properties (Figure S28b, Supporting Information). Therefore, a dry annealing temperature of 60 °C was determined to be optimal. These conditions ensured uniform annealing, enhanced intermolecular interactions, and the production of tough hydrogels with reliable mechanical properties.

### Mechanical and Electrical Characterizations

The mechanical properties of the samples were determined using a Mark‐10 mechanical testing system to obtain stress‐strain curves. Stress was calculated by dividing the applied force by the cross‐sectional area of the specimen, while strain was calculated by dividing the change in length by the original length. The elastic modulus was derived from the initial linear slope of the stress‐strain curve, and toughness was obtained by integrating the area under the stress‐strain curve. Dissipated energy was estimated from the area enclosed by the stress‐strain curves. For electrical testing, the resistance of the samples was measured using a source meter (Keithley 2400; Tektronix, Inc.) Gauge factor (GF) is calculated by the following Equation (1):

(1) $$GF=\left(\Delta R/R_{0}\right)/\varepsilon$$

where R_0_, ΔR, and ε are the initial resistance before stretching, difference of resistance under stretching, and applied strain, respectively. To prepare for mechanical and electrical tests under different conditions, samples underwent varied environments. Specifically, they were exposed to temperatures of either −20 or 70 °C, subjected to a vacuum of 12 psi for 12 h, and stored in the air for one month immediately before conducting the experiments at room temperature.

### Structural Analysis

Hydrogels underwent a 48‐h lyophilization process with a Scientific Pro Freeze Dryer (HR7000‐M; Harvestright, LLC.). Their structural intricacies were examined using a high‐resolution SEM (S‐4800; Hitachi, Inc.). The number of pores and their size were analyzed by using an adapted open‐source MATLAB script.

### Fourier‐Transform Infrared (FTIR) Spectroscopy

FTIR spectroscopy (Thermo Nicolet iS50) was performed in absorption mode with a scan range of 500 – 4000 cm^−1^, 16 scan acquisitions, and a resolution of 4 cm^−1^. Samples were placed on the measuring area that covers the whole surface of the FTIR crystal.

### X‐Ray Diffraction (XRD) Analysis

An XRD (Panalytical Empyrean) was used to characterize the crystallinity and crystallite size of the hydrogels under Cu Kα radiation at 45 kV and 40 mV. The crystallinity of samples is calculated by the ratio of the sum of the deconvoluted crystalline part over the sum of the crystalline and the amorphous deconvoluted parts. The crystallite size of the hydrogels was calculated from the Scherrer's Equation (2):

(2) $$D=\frac{K\lambda }{\left(FWHM\right)\times cos\theta }$$

where D is crystallite size, K is the equipment constant with a value of 0.89, and λ is the wavelength of X‐ray (1.54178 Å). FWHM represents the full width at half maxima of the peak and θ is the diffraction angle.

### Differential Scanning Calorimetry (DSC) Analysis

DSC measurements were conducted on DSC 8500 (Perkin Elmer, USA) with a scanning rate of 10 °C min^−1^ under a nitrogen atmosphere.

### Rheology

A rheometer (Discovery HR‐2; TA Instruments, Ltd.) was used to measure the rheological properties of the inks using a parallel plate geometry (20 mm diameter). The viscosity measurements were carried out with a shear rate range of 0.01 to 1000 s^−1^. Storage modulus and loss modulus as a function of shear stress from 0.77 to 7.7 Pa and 1 to 631 Pa were measured at a constant frequency of 1.5 rad s^−1^ for RH and SH ink, respectively. RH ink required maintaining low shear stress to prevent it from spinning off the plate. All rheological measurements were performed at room temperature. The yield stress was determined as the point where the storage modulus intersects with the loss modulus.

### Free Falling Testing

An experimental setup was established to measure the rebound ratio (r) of the ball under different falling heights (h). A polished steel ball of mass of 11.6 g was placed at heights of 30, 45, and 60 cm above the substrate, respectively. The events were captured using a sample frequency of 240 frames per second for video analysis. To determine the rebound ratio, calculated by the ratio of rebound height (r) to start height (h), a video analysis code (Movie S7, Supporting Information) was developed using the open‐source video libraries for Python (OpenCV). For the toughness demonstration of synthetic webs, the steel ball was dropped with mass of 225, 371, and 534 g at a height of 7 cm. For safeguard demonstration, a flat glass was covered with the R‐Hydrogel, S‐Hydrogel, and D‐Hydrogel with a thickness of 0.5 mm, and observed the impact of a weight of 50 g falling from a height of 15 cm. For the beaker protection demonstration, a beaker was dropped from a height of 45 cm onto the surface of the D‐Hydrogel of 11 × 8 × 0.1 cm^3^.

### Finite Element Analysis (FEA) Analysis

The deformation behavior of hydrogels against a free‐falling steel ball was analyzed using the commercially available software ABAQUS. The hydrogels were modeled with a spider‐web structure, replicating a thickness of 1.25 mm as measured from actual samples. Mechanical properties of the hydrogels were defined using a hyperelastic material model, informed by uniaxial tensile test data. A Poisson's ratio of 0.45 was assigned to the hydrogel material. For the steel ball, the elastic modulus, Poisson's ratio, and density were set as 200 GPa, 0.3, and 8.16 g cm^−^^3^, respectively. The ball, with a diameter of 36.7 mm, was subjected to a free‐fall simulation using a Dynamic Explicit step to capture the interaction with the hydrogel.

### Motion Detection Testing

All the human studies were conducted in compliance with the university regulations and approved by the Institutional Review Board (IRB protocol #: 202212‐009‐03). Silicone cover stranded‐core wires (adafruit, Inc.) and Cu tapes (CST5) were attached to both sides of samples to serve as electrical connectors. The samples were then attached on the finger using Tegaderm Film (3M, Inc.)

### Skin Irritation Testing

Hyperspectral line‐scan images (hypercube) of human skin were captured to investigate inflammation, typically characterized by erythema and alterations in hemoglobin concentrations, following skin irritation. Imaging was facilitated by a monochrome camera (GS3‐U3‐120S6M‐C; FLIR) equipped with a 23 µm wide slit and a groove density of 150 mm^−1^. Illumination was provided by an LED light source with a color temperature of 6,500K (D65). Spectrograph calibration was accomplished using a xenon light source, which emitted multiple narrow peaks at specific wavelengths. A fixed focal length lens (MVL25M1; Navitar) was primarily utilized to image the skin, achieving a field of view as small as 10 mm × 10 mm. RGB images of the same area were taken using a smartphone camera (iPhone 11 Pro; Apple). The D‐Hydrogel was applied to the medial antebrachial cutaneous region of the forearm for ≈10 min, while a 3M tape was attached to the same area for 10 min as a positive control. Images were captured before and after the experiment to enable a comparison of hemoglobin content. A mechanical linear scan step was executed at 0.25 mm increments, and data was collected using a custom MATLAB interface. To extract vital hemodynamic parameters from the hyperspectral image, a tissue reflectance spectral model was applied. Light propagation in the tissue was modeled using the theory of radiative transport and robust approximations such as diffusion, Born, and empirical modeling. The intensity reflected from a biological sample can be represented as a function of λ in the visible range.

## Conflict of Interest

The authors declare no conflict of interest.

## Supporting information



Supporting Information

Supplemental Movie 1

Supplemental Movie 2

Supplemental Movie 3

Supplemental Movie 4

Supplemental Movie 5

Supplemental Movie 6

Supplemental Movie 7

## Data Availability

The data that support the findings of this study are available from the corresponding author upon reasonable request.
