# Supplementary material for: Spider Silk‐Inspired Conductive Hydrogels for Enhanced Toughness and Environmental Resilience via Dense Hierarchical Structuring
Source: Adv Sci (Weinh). 2025 Feb 4;12(12):2500397. doi: 10.1002/advs.202500397 (PMC11948067; doi:10.1002/advs.202500397)
Supplement: Supplementary file 1 — Supporting Information [file ADVS-12-2500397-s005.docx]

Supporting Information

Spider silk-inspired conductive hydrogels for enhanced toughness and environmental resilience via dense hierarchical structuring

*Seokkyoon Hong, Jiwon Lee, Taewoong Park, Jinheon Jeong, Junsang Lee, Hyeonseo Joo, Juan C. Mesa, Claudia Benito Alston, Yuhyun Ji, Sergio Ruiz Vega, Cristian Barinaga, Jonghun Yi, Youngjun Lee, Jun Kim, Kate J. Won, Luis Solorio, Young L. Kim, Hyowon Lee, Dong Rip Kim*, Chi Hwan Lee**

This PDF file includes:

Figure S1 to S28

Table S1

Other Supplementary Materials for this manuscript include the following:

Movies S1 to S7


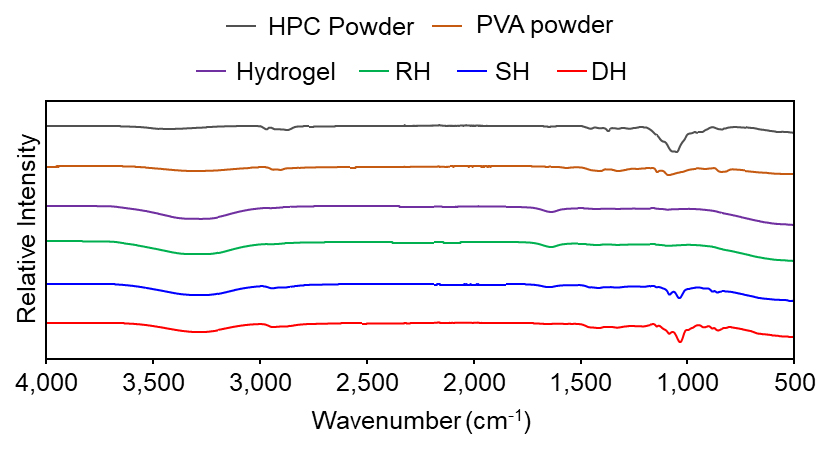


**Figure S1.** FTIR of PVA powder, HPC power, hydrogel, RH, SH, and DH.


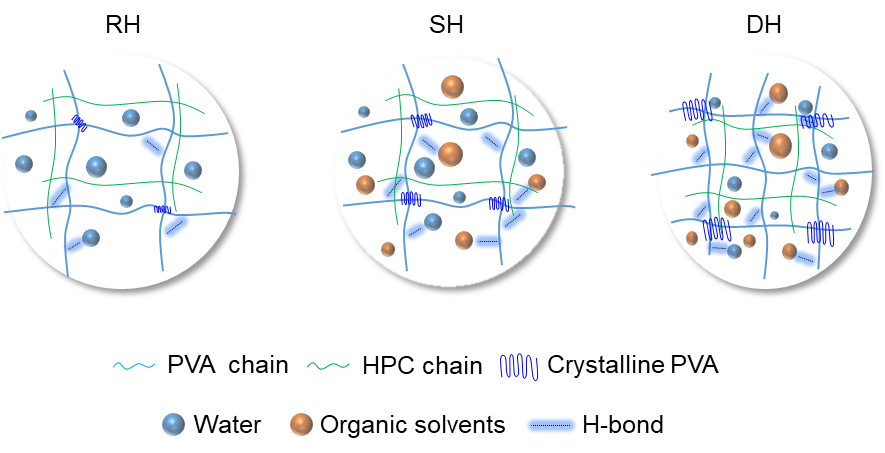


**Figure S2.** Schematics of the R-Hydrogel (RH), S-Hydrogel (SH), and D-Hydrogel (DH).


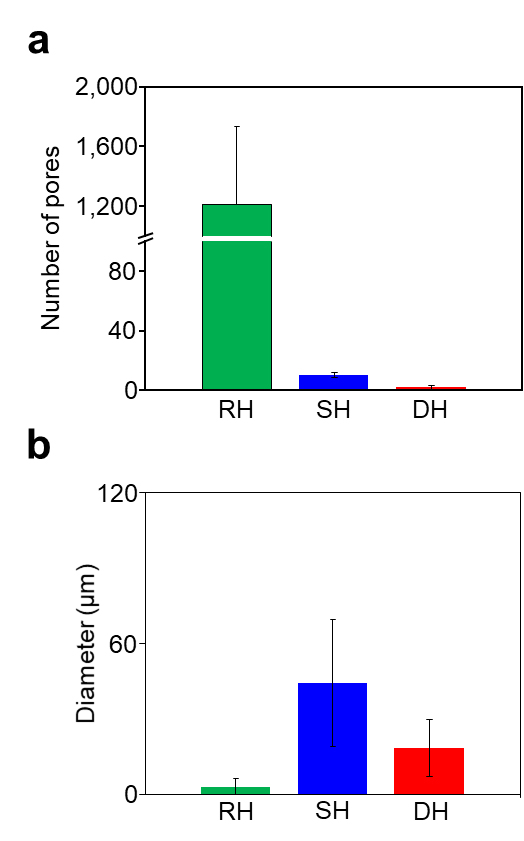


**Figure S3.** a) The number of pores in RH, SH, and DH. b) Pore diameter of RH, SH, and DH.


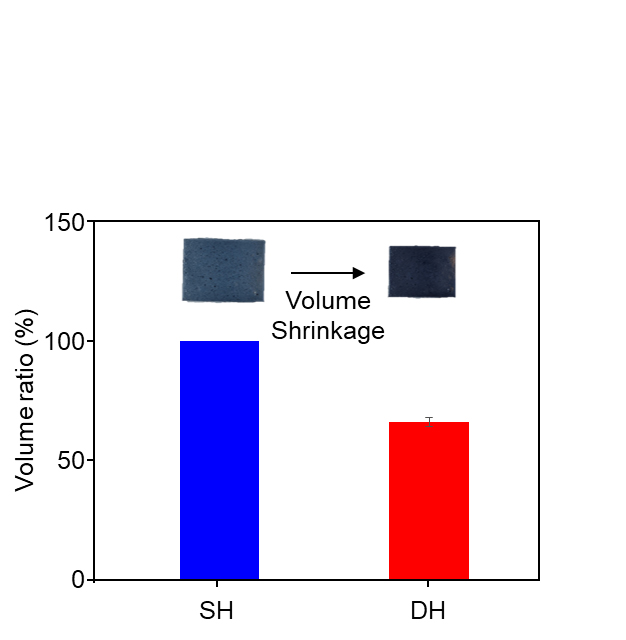


**Figure S4.** Volume shrinkage of SH after the dry annealing.


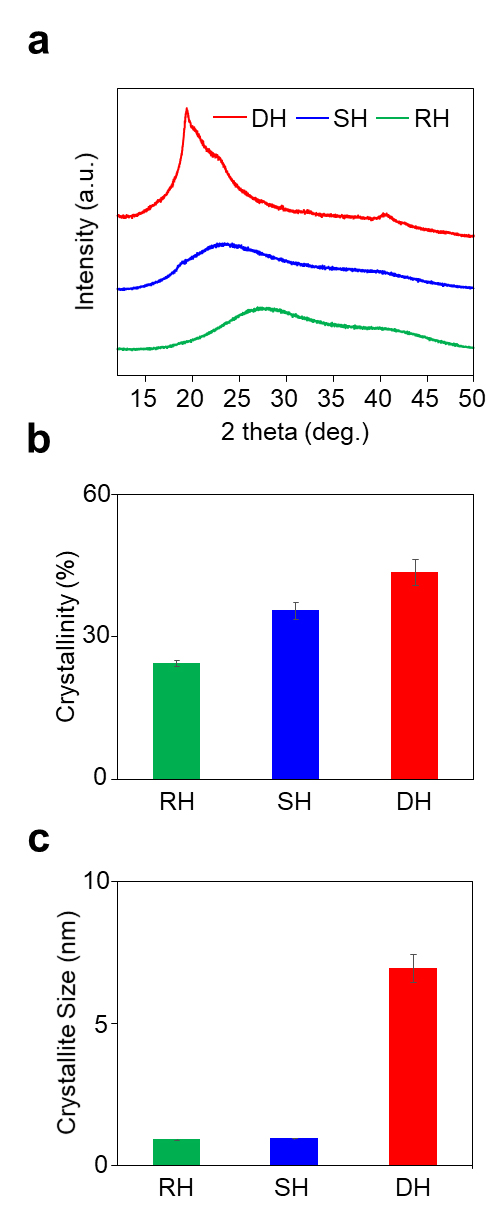


**Figure S5.** Nano-scale analysis. a) XRD analysis of RH, SH, and DH. b) Crystallinity of RH, SH, and DH. c) Crystallite size of RH, SH, and DH.


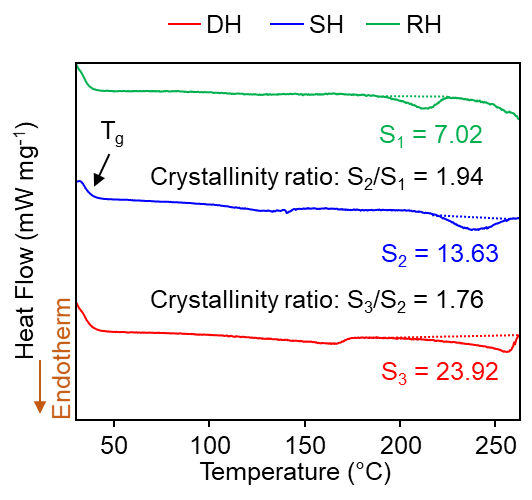


**Figure S6.** Nano-scale analysis. DSC analysis of RH, SH, and DH.


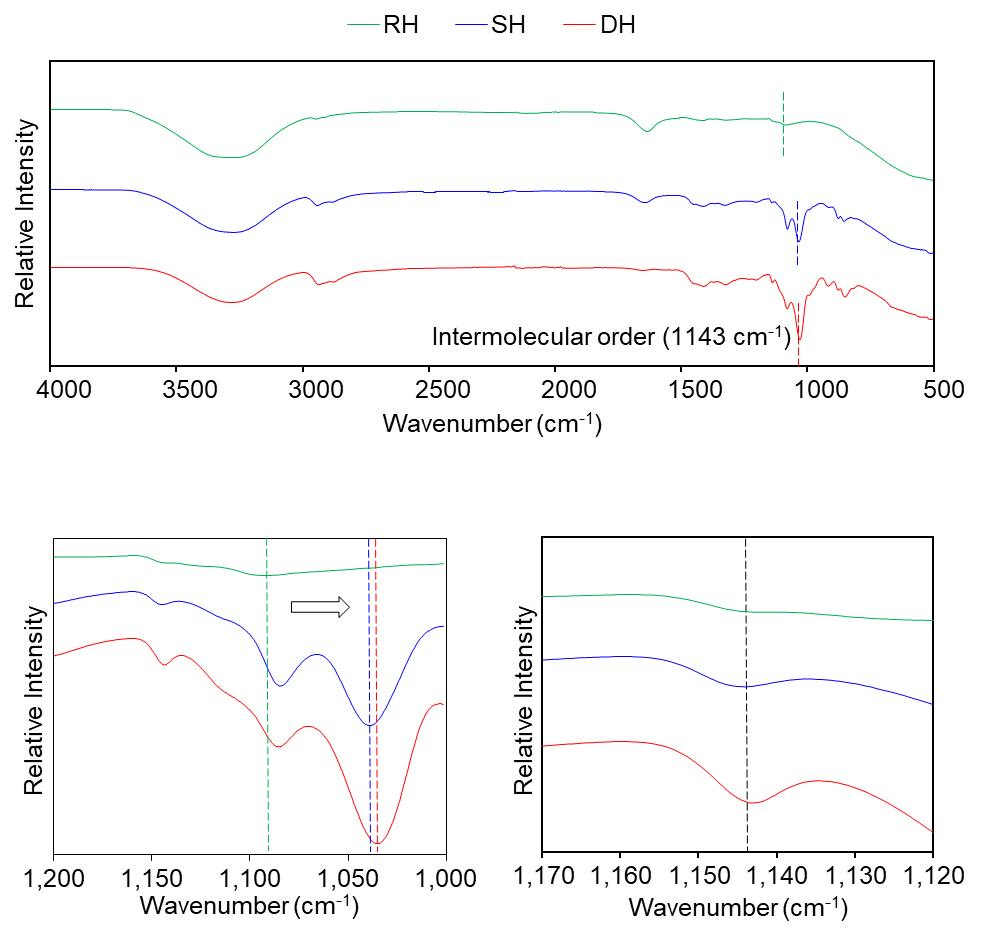


**Figure S7.** Molecular-scale analysis. FTIR spectra of RH, SH, and DH.


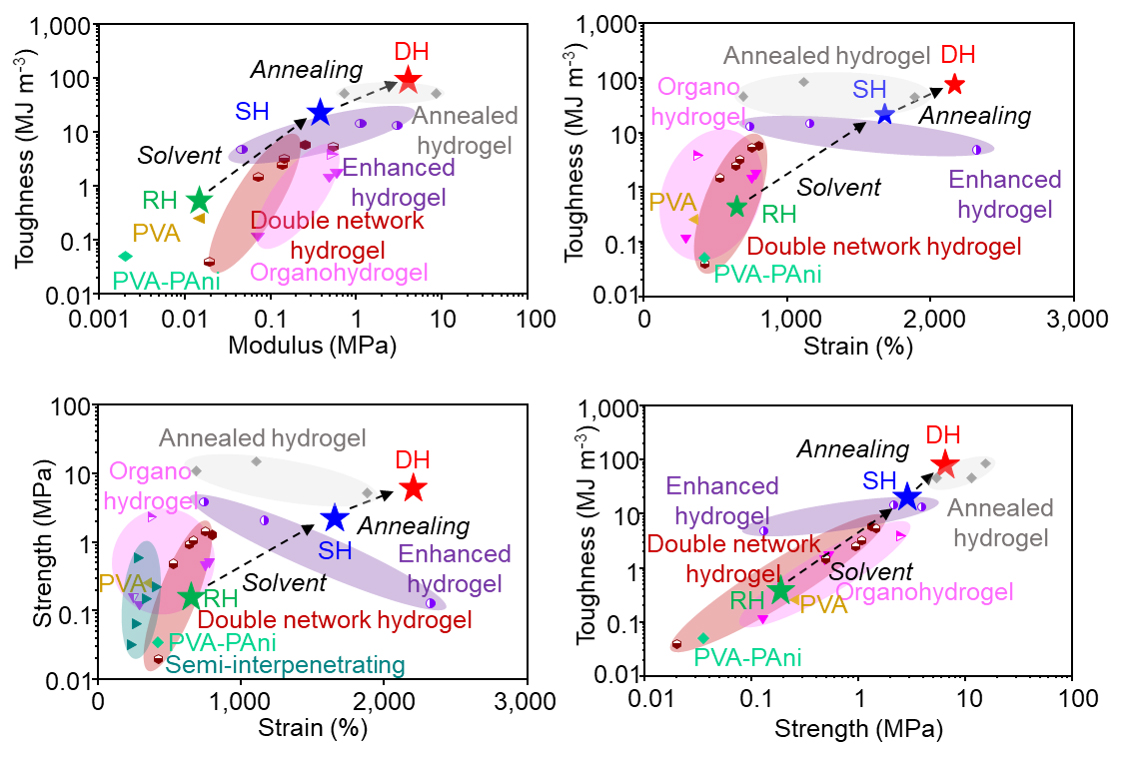


**Figure S8.** Comparison of mechanical properties, including toughness, modulus, strength, and strain of RH, SH, and DH with those from previous studies.


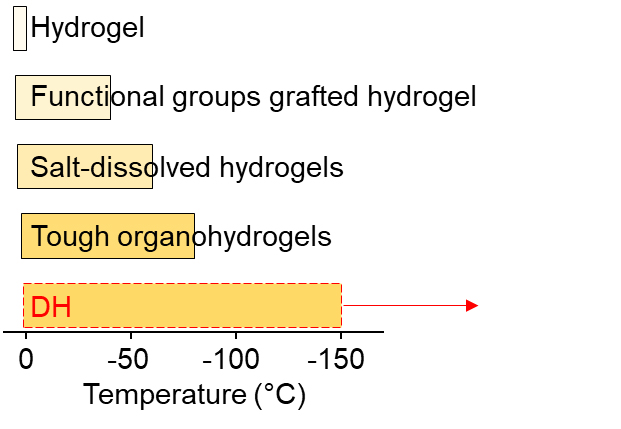


**Figure S9.** Comparison of freezing resistance performance of DH with that of previous studies.


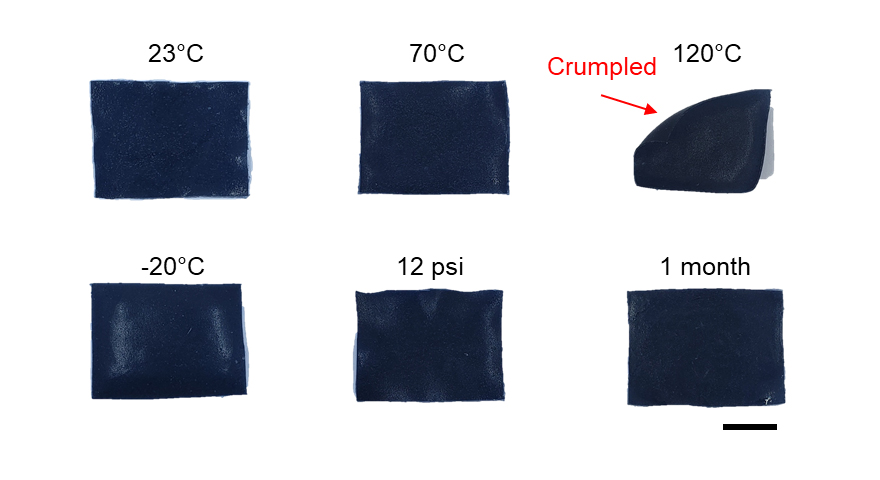


**Figure S10.** Environmental tolerance of DH. Images of DH under various conditions. Scale bar, 2 cm.


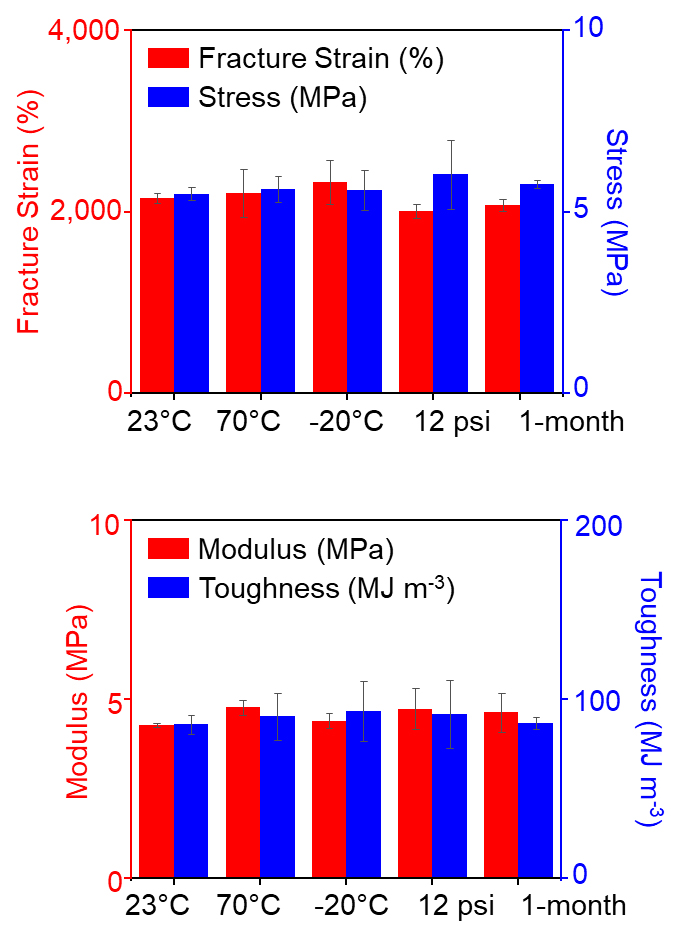


**Figure S11.** Mechanical properties including modulus, toughness, fracture strain and stress of DH under various environment.


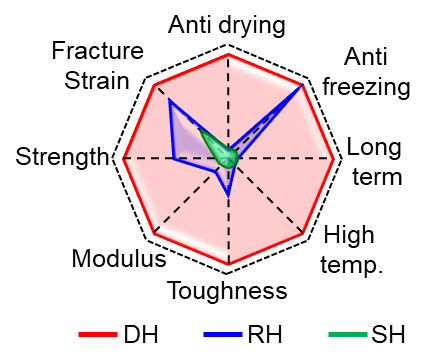


**Figure S12.** Capability radar diagram of the comprehensive performances including mechanical properties and environmental stability of RH, SH, and DH.


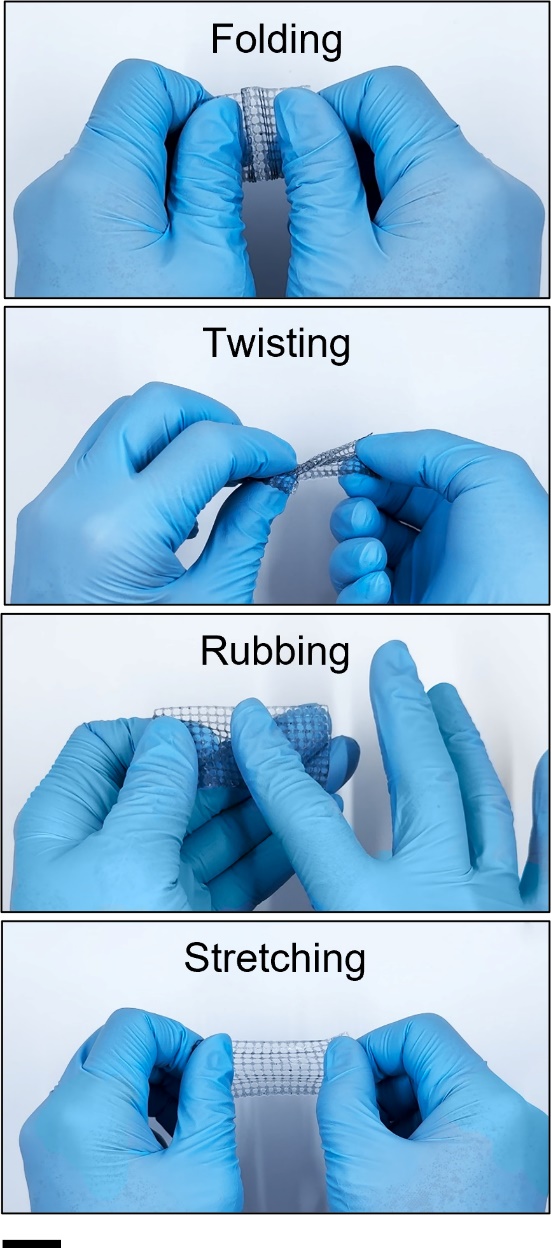


**Figure S13.** Images of a printed DH under folding, twisting, rubbing, and stretching. Scale bar, 2 cm.


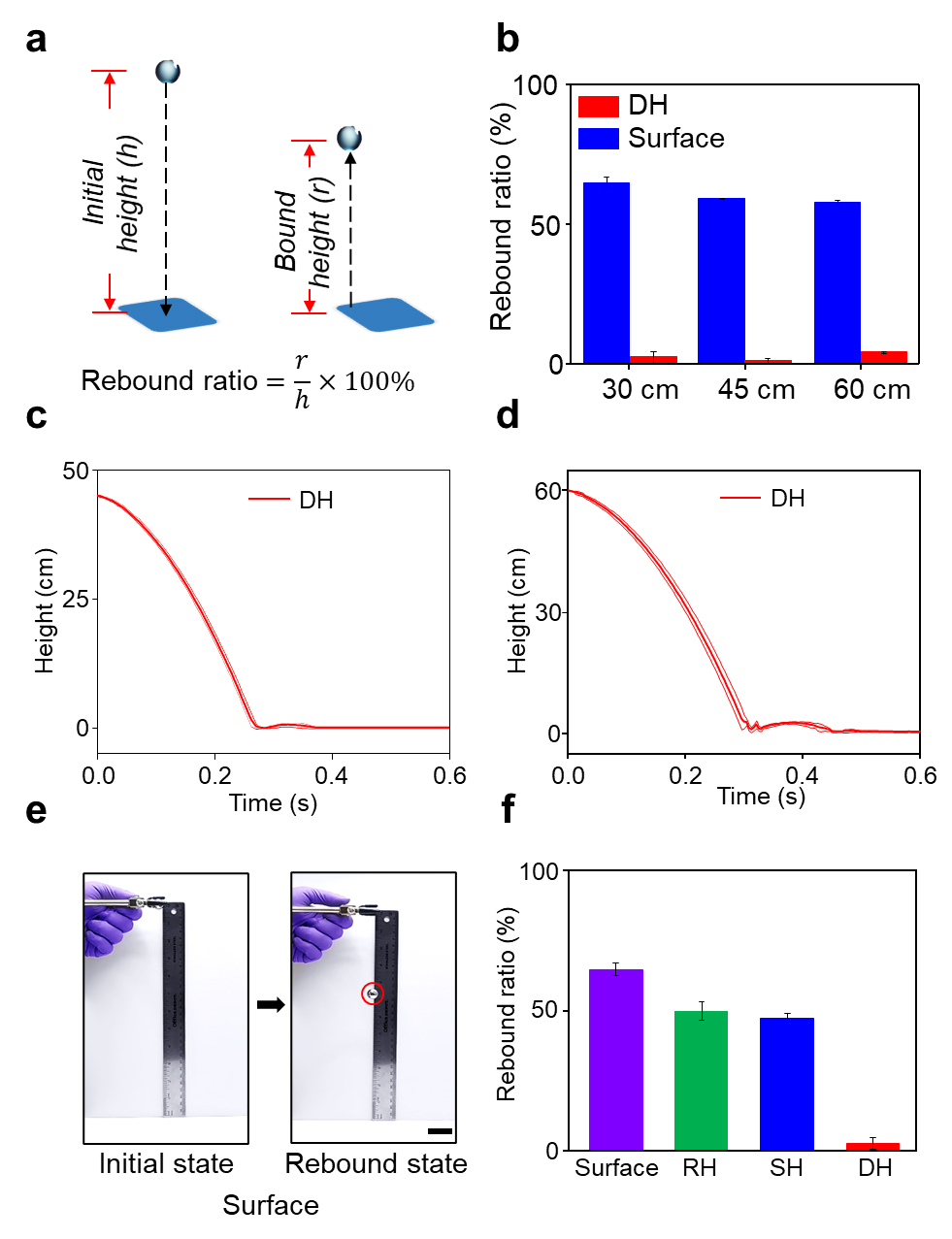


**Figure S14.** Free-falling experiment. a) Schematic of free-falling experiment. b) Rebound ratio of the steel ball onto DH and rough surface from different heights. Height versus time for steel-ball drop and rebound trails on DH with initial heights of c) 45 cm and d) 60 cm. e) Images of free-falling experiment, showing the rebounding of a steel ball onto rough surface. Scale bar, 3 cm. f) Rebound ratio of the steel ball onto rough surface, RH, SH, and DH.


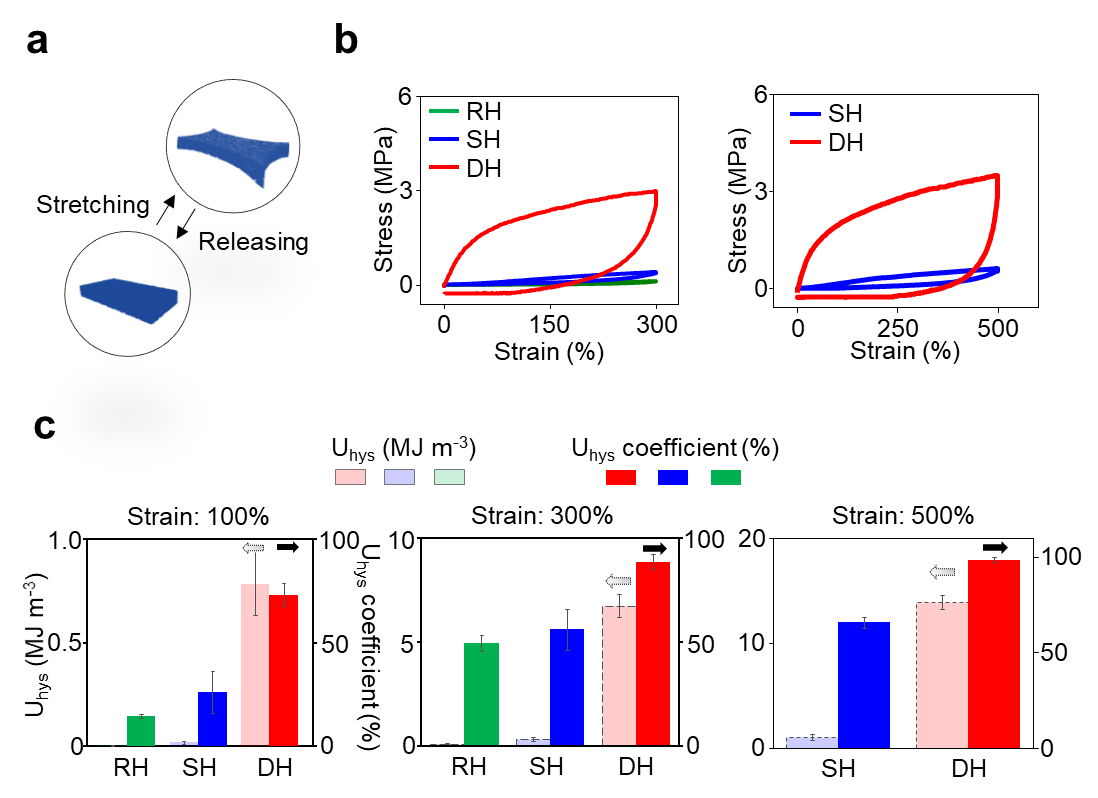


**Figure S15.** Energy dissipation. a) Schematic of DH under stretching and releasing. b) Loading-unloading curves of RH, SH, and DH at tensile strains of 300% and 500%. c) Dissipation energy and energy dissipation coefficient of RH, SH, and DH at strains of 100%, 300%, and 500%.


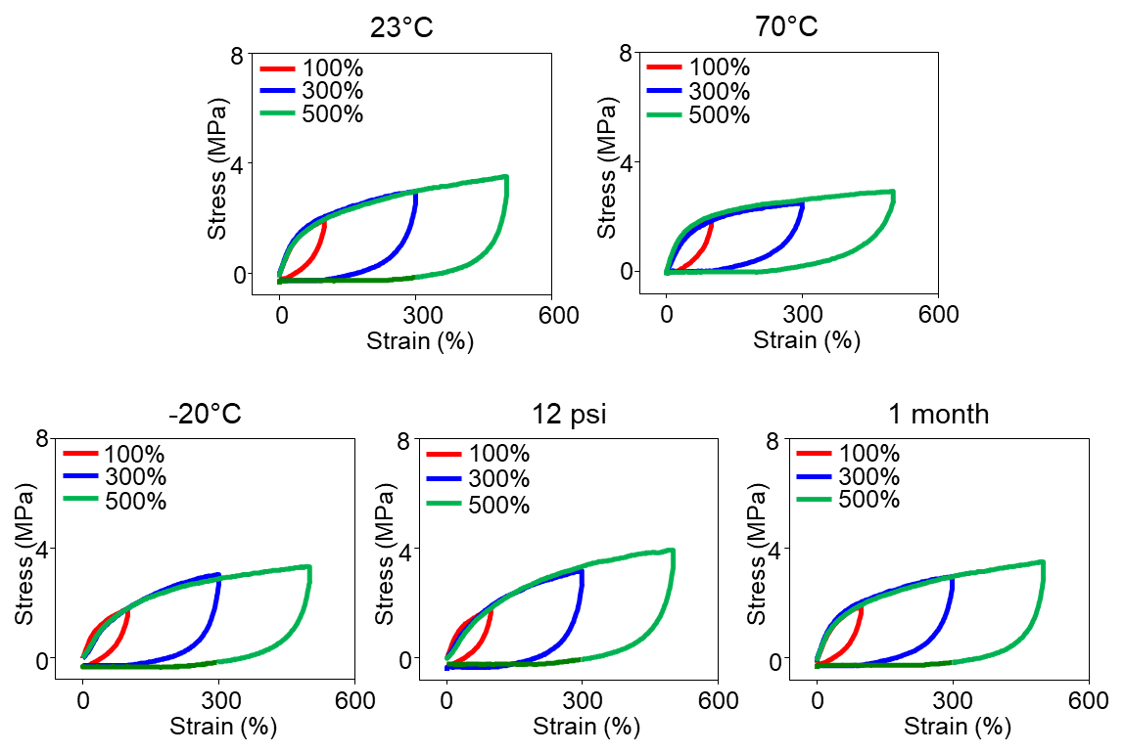


**Figure S16.** Stress-strain curves of DH at strains of 100%, 300%, and 500% under various conditions.


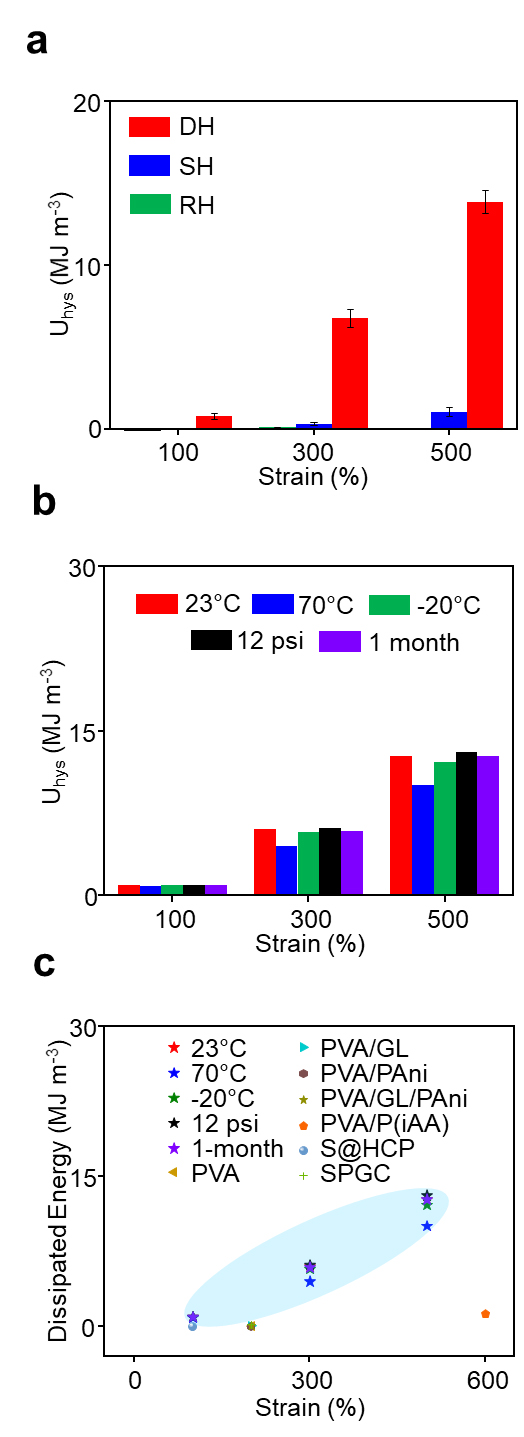


**Figure S17.** a) Energy dissipation of RH, SH, and DH at strains of 100%, 300%, and 500%. b) Energy dissipation of DH at strains of 100%, 300%, and 500% under various conditions. c) Comparison of strain and dissipated energy of DH with those from previous studies.


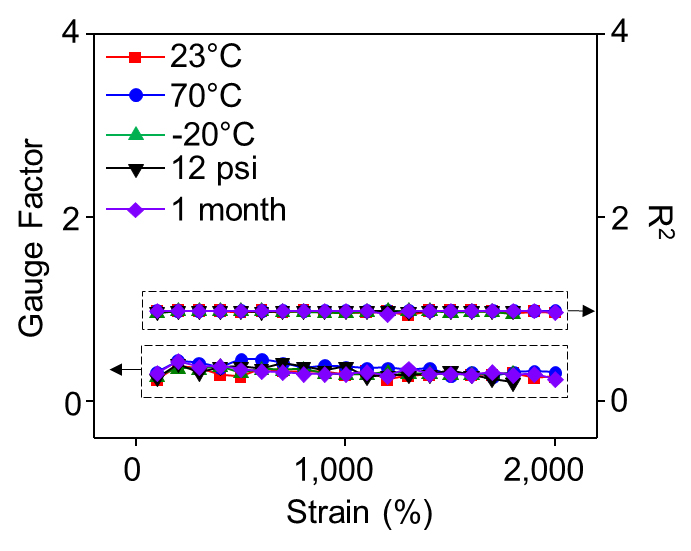


**Figure S18.** Gauge factor and linearity of DH up to a strain of 2,000%.


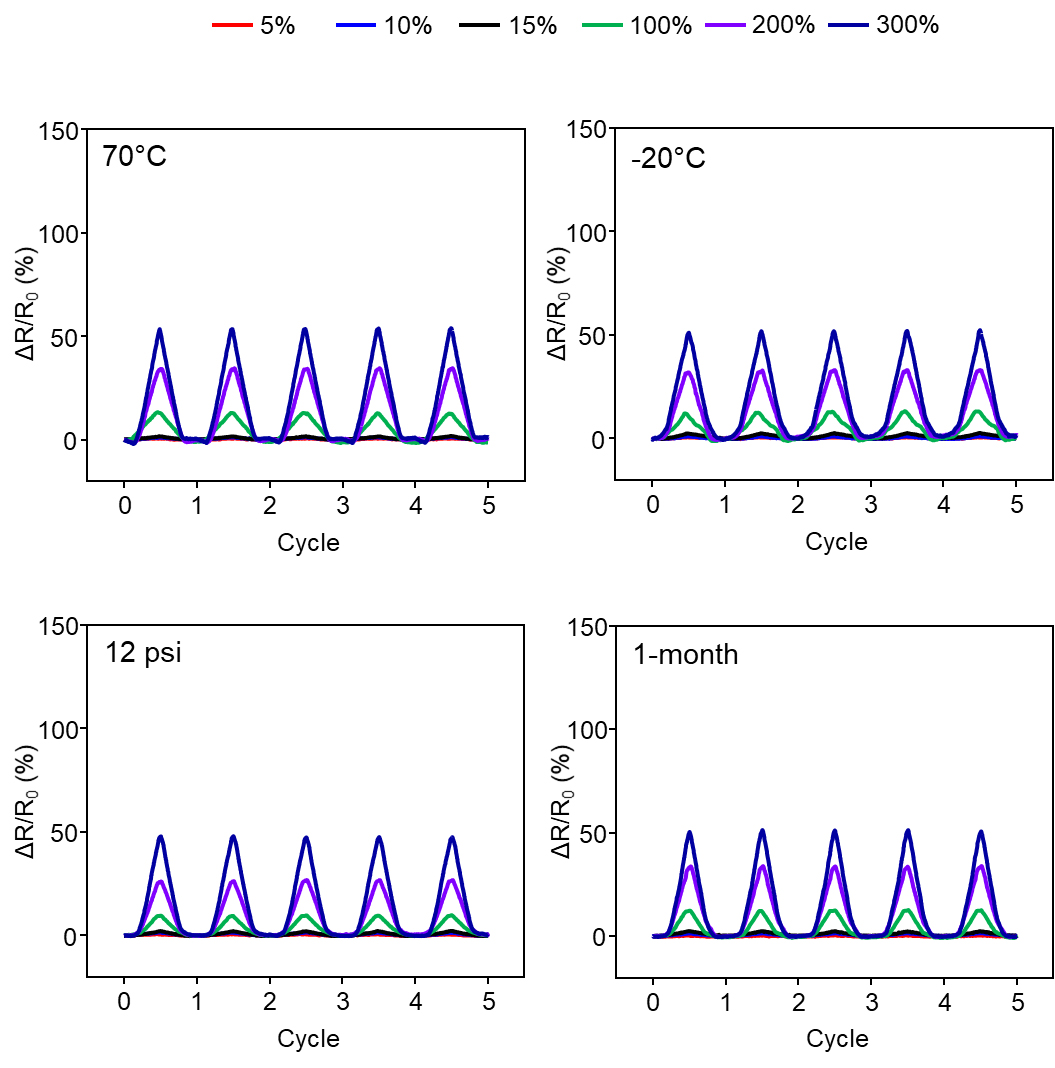


**Figure S19.** ∆R/R_0_ of DH under loading-unloading cycles at strains of 5%, 10%, 15%, 100%, 200% and 300% under various environments.


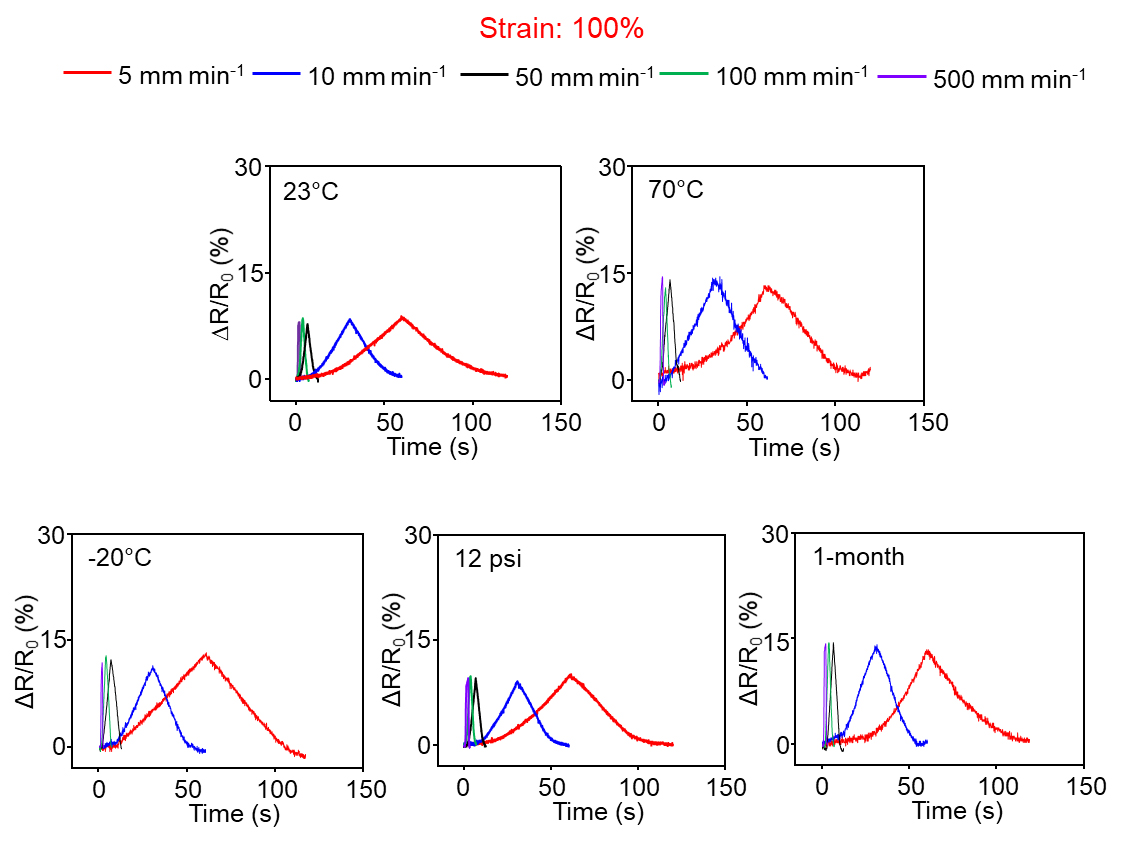


**Figure S20.** ∆R/R_0_ of DH under various test rates from 5 to 500 mm min^−1^ at a strain of 100% under diverse conditions.


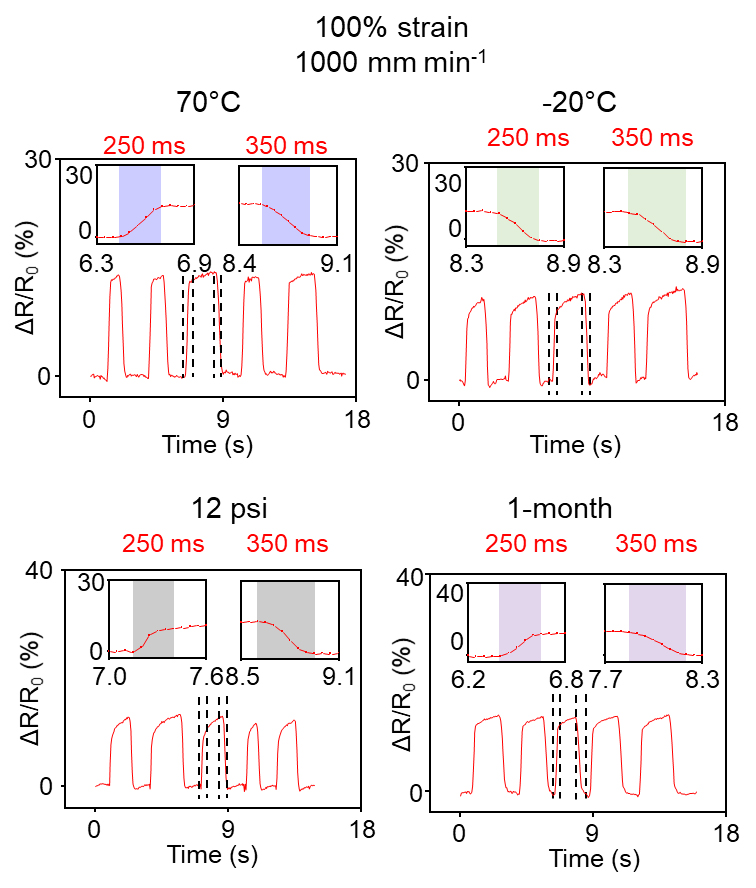


**Figure S21.** Response/recovery time of DH at a strain of 100% under various conditions.


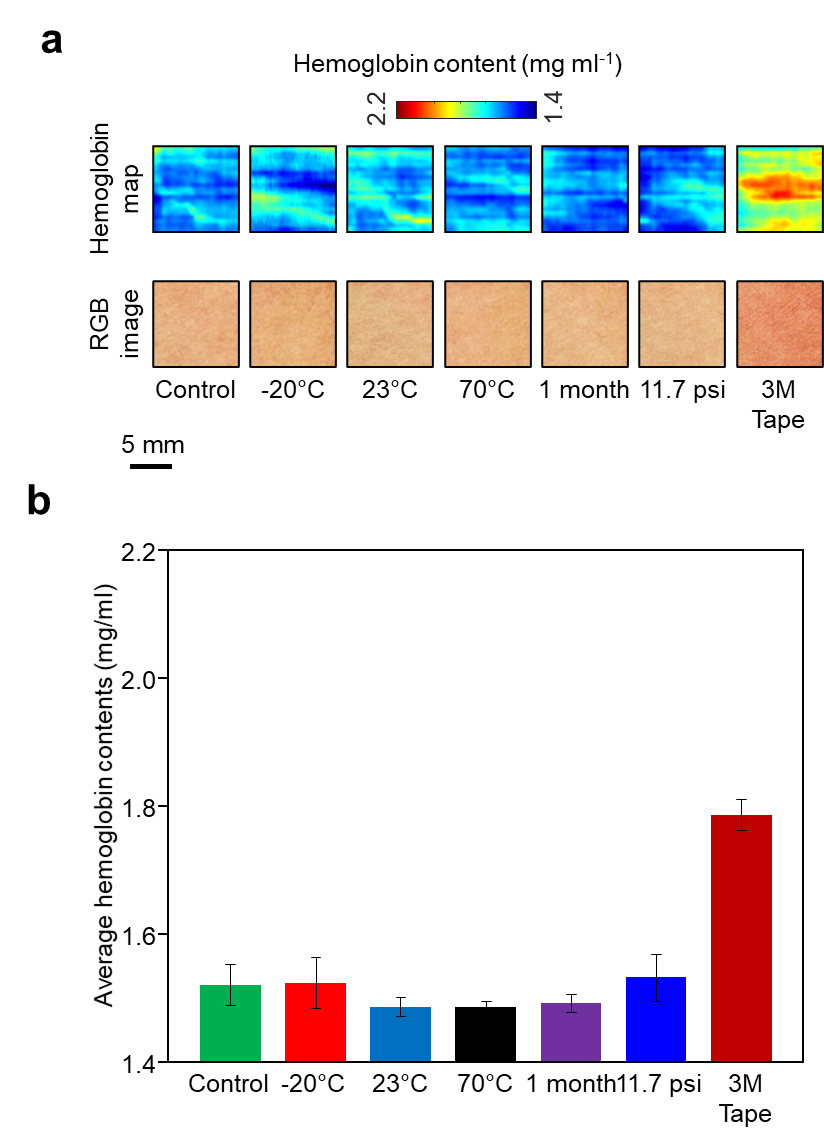


**Figure S22.** Skin irritation. a) RGB images of the forearm skin before (control) and after application of DH, compared to the skin after applying 3M Tape. The below column shows the hemoglobin map measured using a hyperspectral line-scanning system. b) Bar graph displaying the average hemoglobin content of a sampled area.


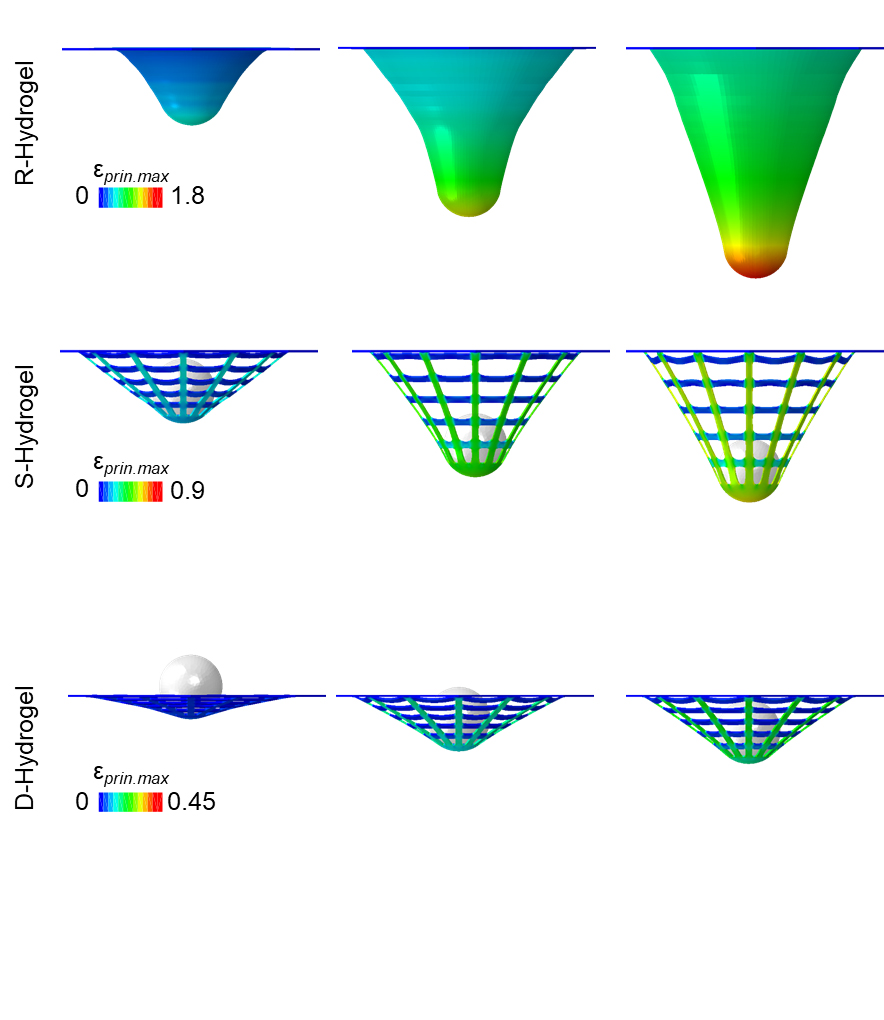


**Figure S23.** FEA results of the RH, SH, and DH under the steel ball impact.


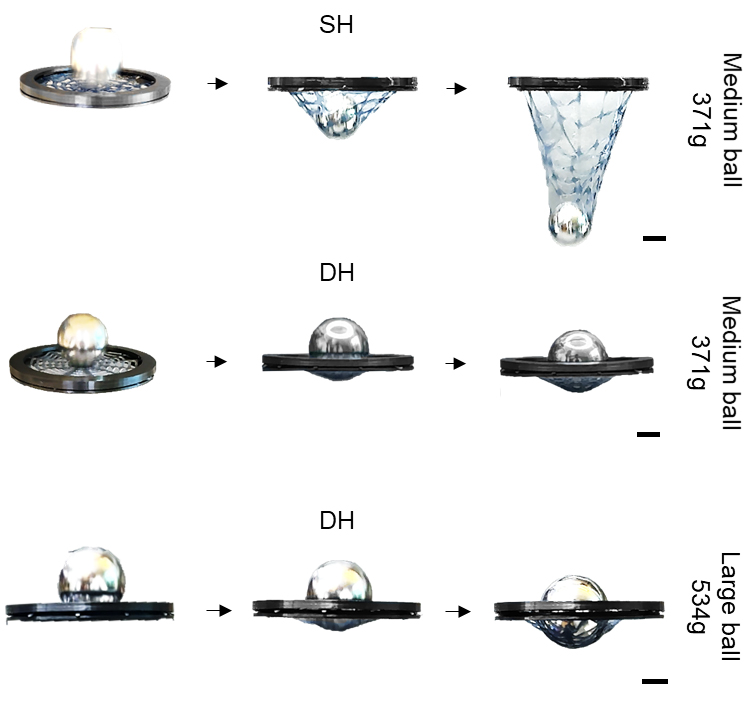


**Figure S24.** Image of SH and DH with different steel-ball weight for toughness demonstration. Scale bar, 2 cm.


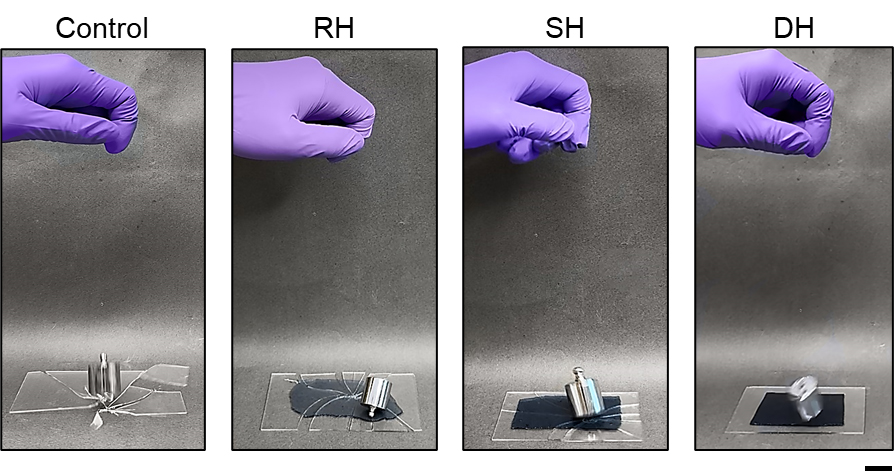


**Figure S25.** Images of the bare glass and RH, SH, and DH used as glass protection materials when subjected to impact. Scale bar, 2 cm.


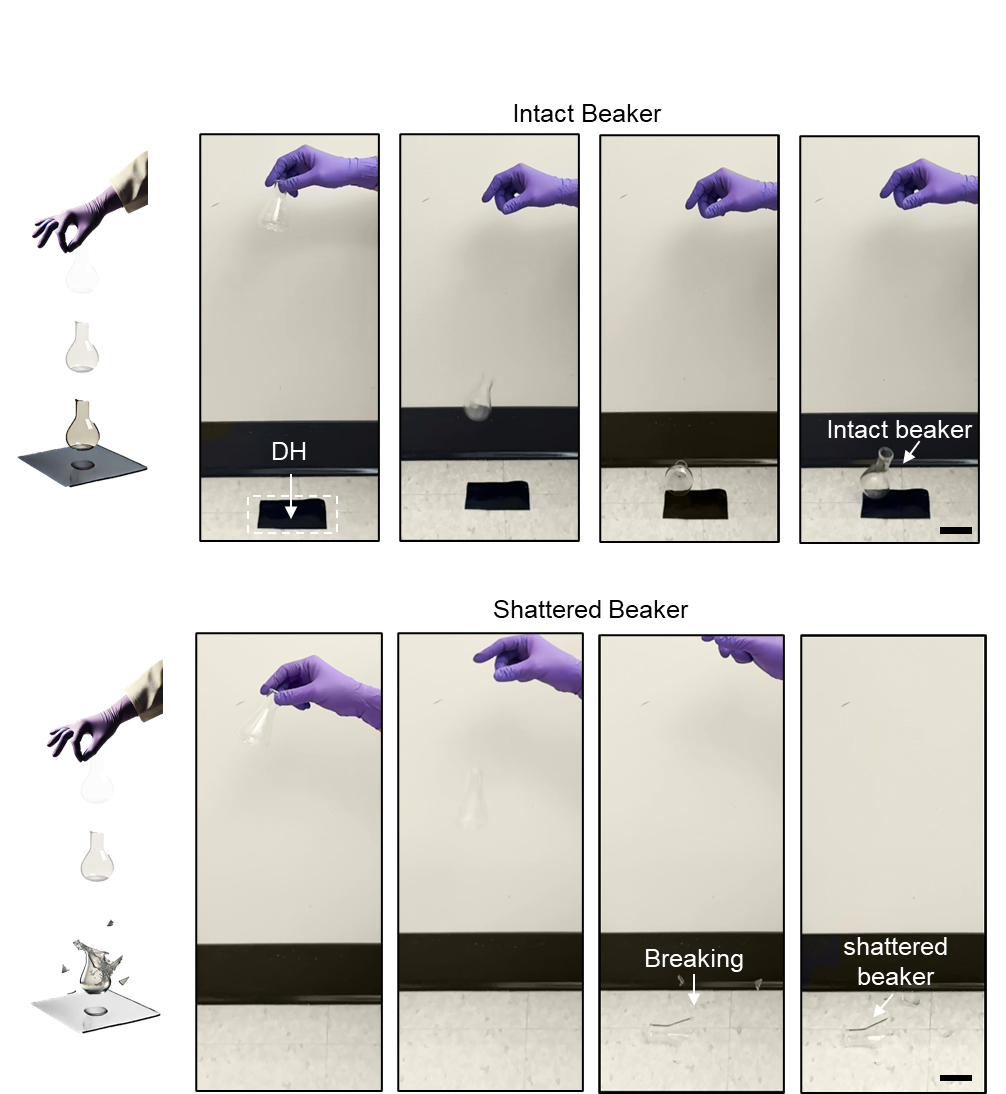


**Figure S26.** Snapshots of beaker, impacting DH and surface. Scale bar, 5 cm.


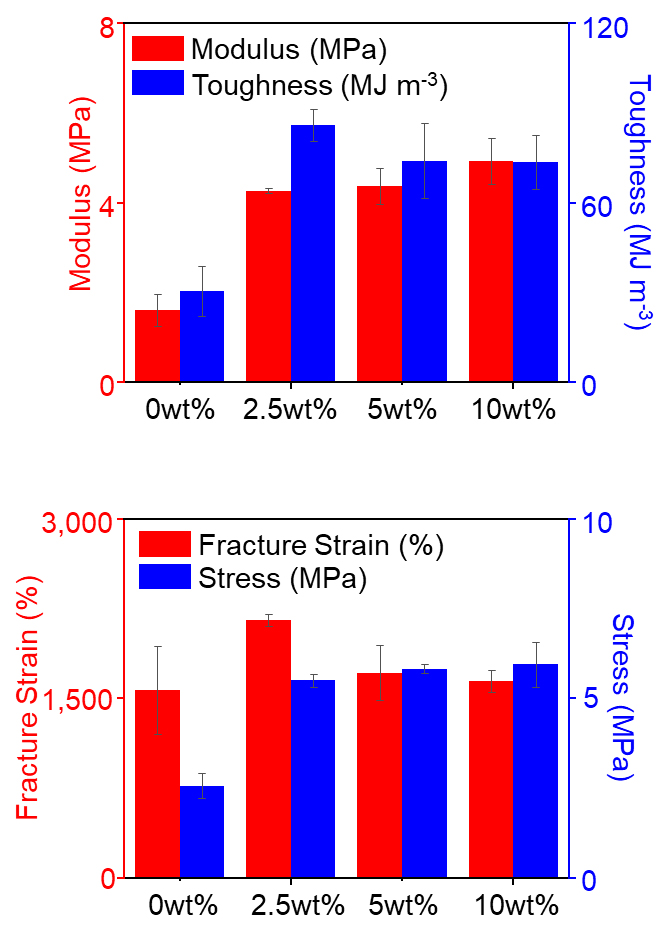


**Figure S27.** Mechanical properties of DH, including modulus, toughness, fracture strain and stress.


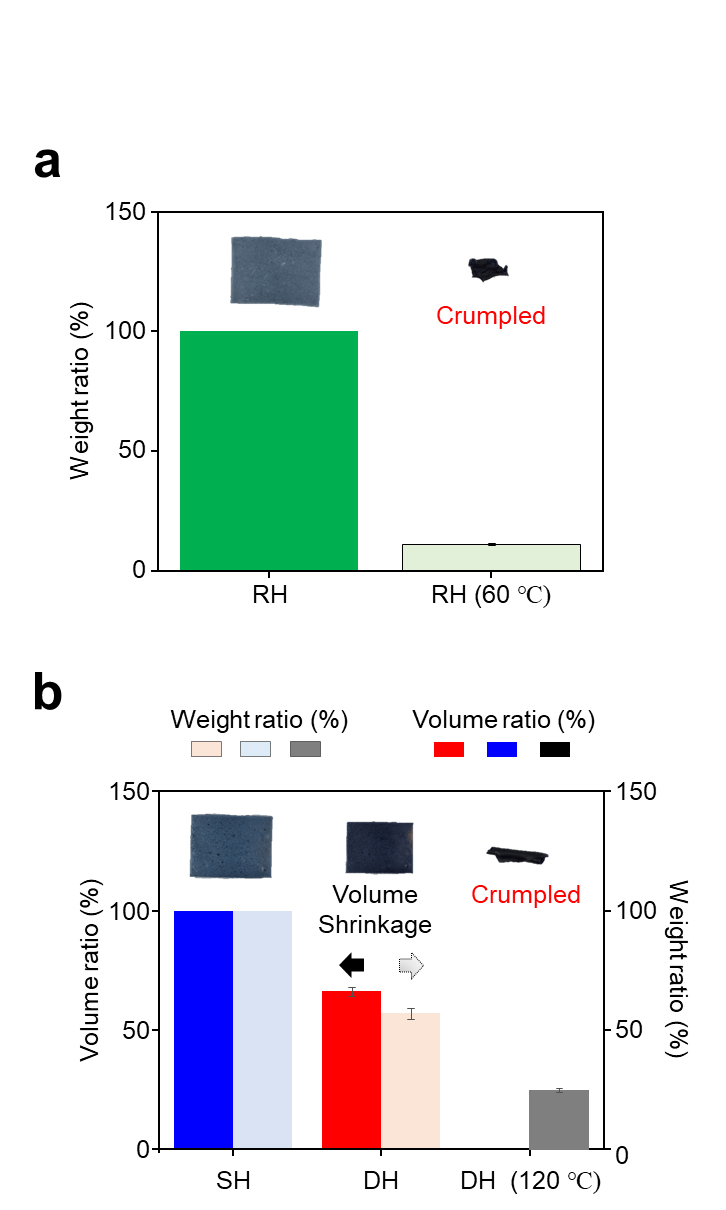


**Figure S28.** a) Weight ratio changes of RH after dry annealing at 60 ℃. Inset: Images of RH after dry annealing at 60 ℃. Scale bar, 2 cm. b) Volume and weight ratio changes of SH after dry annealing at 60 ℃ and 120 ℃. Inset: Images of SH after dry annealing at 60 ℃ and 120 ℃. Scale bar, 2 cm.


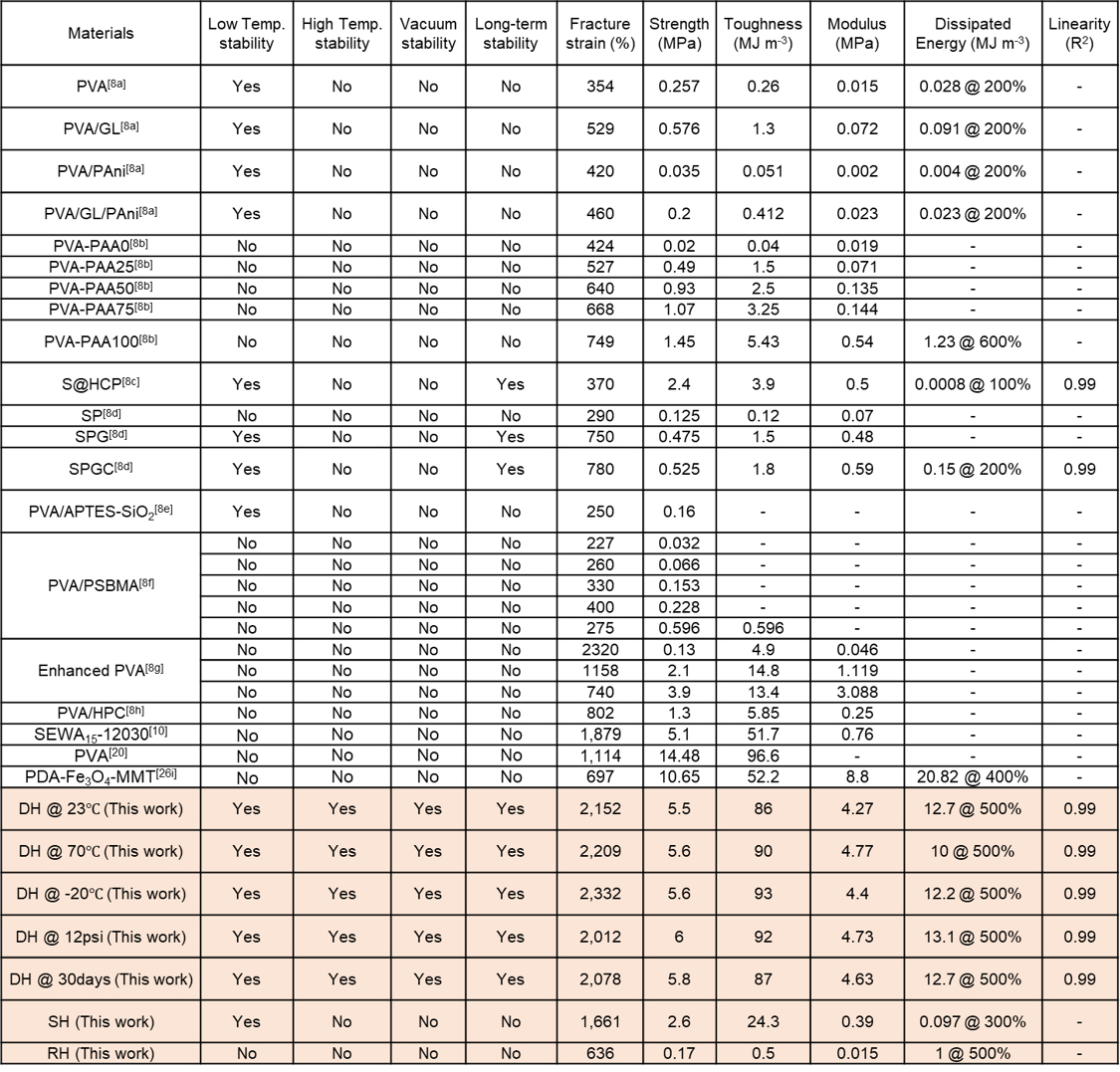


**Table S1.** Performance comparison of RH, SH, and DH with previous studies.

**Movie Captions**

**Movie S1.** D-Hydrogels, supporting a 70 kg man.

**Movie S2.** A printed D-Hydrogel under twisting, folding, rubbing, and stretching.

**Movie S3.** Free-falling experiments on the bare surface, R-Hydrogel, S-Hydrogel, and D-Hydrogel.

**Movie S4.** Impact of steel ball on R-Hydrogel film, synthetic webs of the S-Hydrogel and D-Hydrogel.

**Movie S5.** Comparison of glass protection between bare glass and glasses covered with R-Hydrogel, S-Hydrogel, and D-Hydrogel.

**Movie S6.** Protection of a beaker from breakage by the D-Hydrogel.

**Movie S7.** A video analysis code for free-falling experiments.
